# Recent Advances in the Label-Free Characterization of Exosomes for Cancer Liquid Biopsy: From Scattering and Spectroscopy to Nanoindentation and Nanodevices

**DOI:** 10.3390/nano11061476

**Published:** 2021-06-02

**Authors:** Riccardo Di Santo, Sabrina Romanò, Alberto Mazzini, Svetlana Jovanović, Giuseppina Nocca, Gaetano Campi, Massimiliano Papi, Marco De Spirito, Flavio Di Giacinto, Gabriele Ciasca

**Affiliations:** 1 Fondazione Policlinico Universitario A. Gemelli IRCCS, 00168 Roma, Italy; riccardo.disanto92@gmail.com (R.D.S.); sabrina.romano@unicatt.it (S.R.); alberto.mazzini01@icatt.it (A.M.); Giuseppina.nocca@unicatt.it (G.N.); massimiliano.papi@unicatt.it (M.P.); flavio.digiacinto@unicatt.it (F.D.G.); 2 Dipartimento di Neuroscienze, Sezione di Fisica, Università Cattolica Del Sacro Cuore, 00168 Roma, Italy; 3 “Vinča” Institute of Nuclear Sciences—National Institute of the Republic of Serbia, University of Belgrade, 11000 Belgrade, Serbia; svetlanajovanovic@vin.bg.ac.rs; 4 Dipartimento di Scienze Biotecnologiche di Base, Cliniche Intensivologiche e Perioperatorie, Università Cattolica del Sacro Cuore, 00168 Rome, Italy; 5 Rome International Centre Materials Science Superstripes RICMASS, via dei Sabelli 119A, 00185 Rome, Italy; gaetano.campi@ic.cnr.it; 6 Institute of Crystallography, CNR, via Salaria Km 29. 300, Monterotondo Stazione, 00016 Roma, Italy

**Keywords:** exosome, extracellular vesicles, liquid biopsies, label-free, biofluids, microfluidics, nanodevice, SAXS, FTIR, AFM, personalized medicine

## Abstract

Exosomes (EXOs) are nano-sized vesicles secreted by most cell types. They are abundant in bio-fluids and harbor specific molecular constituents from their parental cells. Due to these characteristics, EXOs have a great potential in cancer diagnostics for liquid biopsy and personalized medicine. Despite this unique potential, EXOs are not yet widely applied in clinical settings, with two main factors hindering their translational process in diagnostics. Firstly, conventional extraction methods are time-consuming, require large sample volumes and expensive equipment, and often do not provide high-purity samples. Secondly, characterization methods have some limitations, because they are often qualitative, need extensive labeling or complex sampling procedures that can induce artifacts. In this context, novel label-free approaches are rapidly emerging, and are holding potential to revolutionize EXO diagnostics. These methods include the use of nanodevices for EXO purification, and vibrational spectroscopies, scattering, and nanoindentation for characterization. In this progress report, we summarize recent key advances in label-free techniques for EXO purification and characterization. We point out that these methods contribute to reducing costs and processing times, provide complementary information compared to the conventional characterization techniques, and enhance flexibility, thus favoring the discovery of novel and unexplored EXO-based biomarkers. In this process, the impact of nanotechnology is systematically highlighted, showing how the effectiveness of these techniques can be enhanced using nanomaterials, such as plasmonic nanoparticles and nanostructured surfaces, which enable the exploitation of advanced physical phenomena occurring at the nanoscale level.

## 1. Introduction

In many clinical situations, cancer diagnosis requires single or repeated tissue biopsies of a suspected cancerous region. This procedure is invasive and often associated with pain, discomfort, and risk for the patients. Additionally, the tissue region that needs to be sampled can be highly heterogeneous, thus leading to ambiguous conclusions, hardly accessible, or even completely inaccessible by surgery. These drawbacks limit the frequencies with which a region can be sampled to check for cancer, thus hindering the possibility to perform accurate diagnoses, especially in the early stages of the pathology. Liquid biopsy offers a promising diagnostic alternative, because it relies on the analysis of biofluids, such as blood, saliva, and urine [1,2,3,4,5,6]. Widely used biomarkers in liquid biopsy include cell-free nucleic acids, such as DNA, mRNA, and miRNA, circulating tumor cells, and extracellular vesicles (EVs), which are nanosized lipid vesicles secreted by most cell types. The identification of cancer-specific material in the latter molecular class suggests these nano-sized EVs to be an attractive platform for biomarker development in the field of liquid biopsy and personalized medicine [7,8,9,10,11,12,13].

In this context, it is important to recognize that EVs are highly heterogeneous in chemical make-up. Three main classes of EVs differing in size can be distinguished, namely, exosomes (EXOs), microvesicles (MVs), and apoptotic bodies (ABs) [14,15,16,17,18]. Aside from size differences, the three EV types can be distinguished because of different biogenic mechanisms.

A large research effort in this area has led to the discovery of a wide number of potential cancer biomarkers, mostly based on EXOs. Notably, EXOs are often shed by tumor cells in higher numbers in comparison to normal cells, because tumorigenesis affects many pathways regulating EV release. A higher EXO concentration is thus associated with increasing tumor mass or severity, making EXO-based biopsy attractive as a prognostic biomarker [1,2,3,4,5,7,8,9,10,11,12,13,19,20,21,22,23].

Despite this huge diagnostic potential, EXOs have still not been widely applied in clinical settings. In this regard, two main motivations can be highlighted: (i) the first related to extraction and purification methods; (ii) the second related to EV characterization and downstream analysis.

Conventional isolation methods mostly rely on time-consuming ultracentrifugation steps and require specialized personnel, working with expensive equipment [24]. Additionally, ultracentrifugation needs large sample volumes and often does not produce high-purity samples. Other purification methods have been developed so far, including size exclusion chromatography, polymer-based precipitation, and immunocapture approaches. On the one hand, these methods avoid the use of expensive equipment; on the other hand, they are plagued by several limitations including long operation times, unknown contaminants in commercial kits, and the problem of often being restricted to EXOs with a single antigen.

Apart from extraction methods, a change in the paradigm of EXO analysis is also required to further stimulate their translational process in diagnostics. Although effective conventional techniques exist for EV characterization, such as Western blotting, ELISA, and omics approaches, these methods have some drawbacks, because they are often qualitative and need extensive labeling or complex sampling techniques that can alter the relative ratio of molecular classes.

**Figure 1:**
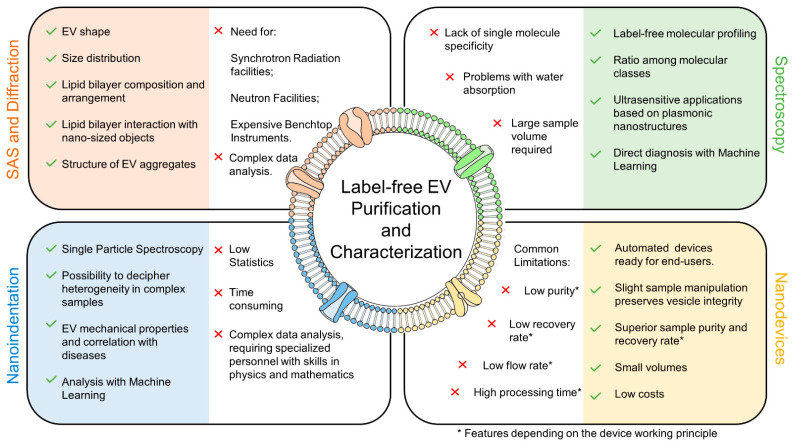
Key characteristics of label-free techniques in EV science.

In this context, novel label-free approaches are rapidly emerging in EV research. These methods include nanodevices for EV purification, and vibrational spectroscopies, scattering, and nanoindentation for EV characterization. The potential advantages and disadvantages of these techniques and their key characteristics in EXO science are summarized in Figure 1.

In this review, we discuss the recent scientific and technological advances in these label-free techniques, highlighting their complementary role compared to conventional and more established methods in EV science. We stress the flexibility of these methods and how this flexibility provides fertile ground for the discovery of novel and unexplored cancer biomarkers in EXO-based liquid biopsy. In this process, the impact of nanotechnology is systematically highlighted, showing how the effectiveness of these techniques can be enhanced using nanomaterials, such as plasmonic nanoparticles and nanostructured surfaces, which enable the exploitation of advanced physical and chemical effects occurring at the nanoscale level.

## 2. Extracellular Vesicles Classification and Biogenesis

**Figure 2:**
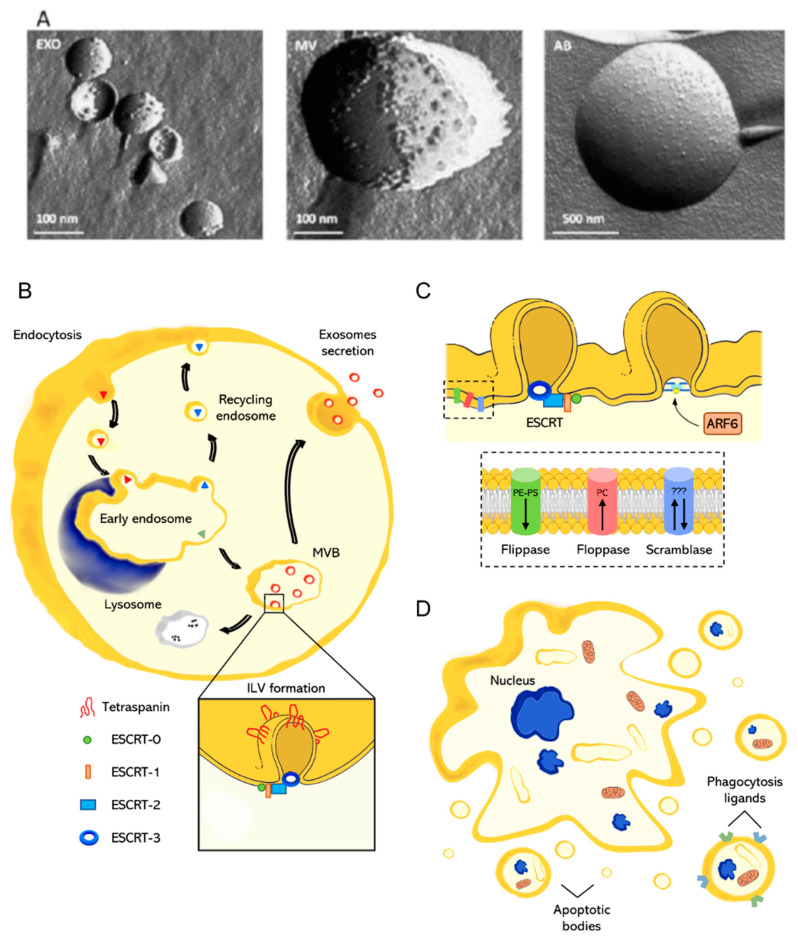
(**A**) EV classification and biogenesis: freeze-fracture transmission electron microscopy of the different EV types, reprinted with permission from ref. [25]. Copyright (2017 Elsevier). (**B**) Mechanisms of biogenesis of EXOs, with emphasis on the role of tetraspanins and ESCRT complexes; (**C**) mechanisms of MV biogenesis, with emphasis on the role of ARF6, ESCRT complexes, flippase, floppase, and scramblase. (**D**) Mechanism of formation of apoptotic bodies.

The classification of the heterogeneous family of EVs has represented a hard task since their discovery. Currently, the scientific community classifies EVs into exosomes (EXOs), microvesicles (MVs), and apoptotic bodies (ABs) [14,15,16,17,18]. This classification mainly relies on the vesicle size [25] (Figure 2A) and biogenesis/secretion mechanism (Figure 2B,D), as summarized in Table 1. EXOs are the smallest subgroup (diameter between 30 and 150 nm) [26], whereas MVs and ABs are larger and more polydisperse, with a reported diameter between 100 and 1000 nm and 100 and 5000 nm, respectively [27,28]. These vesicle size ranges overlap to some extent. The mechanism of biogenesis remains the leading distinction among the subgroups. EXOs originate from the endosomal compartment, a collection of membranous organelles for intracellular sorting. MVs derive from the outward budding of the plasma membrane and, as EXOs, play an important role as intercellular mediators in both physiological and pathological processes. Differently, ABs are generated due to cell apoptosis when cytoskeleton fragmentation causes the plasma membrane to swell outward.

### 2.1. Exosomes

The formation of EXOs is a process occurring within the endosomal pathway (Figure 2B). Extracellular molecules internalized by cells are packaged into endocytic vesicles which fuse and pour out their content in early endosomes. At this point, material due to be recycled (e.g., membrane proteins, receptors) return to the plasma membrane into recycling endosomes, while material due for lysosomal degradation or exocytosis follows a different path along with the transformation of early endosomes into late endosomes [29]. This transformation includes modifications of the endosomal environment together with protein and lipid remodeling [30,31]. Above all, acidification mediated by the proton pump V-ATPase is a key step for endosome maturation that controls several processes (i.e., receptor–ligand dissociation, movement across the microtubule network, enzyme activity) [32]. During this transformation, molecules are sorted into small vesicles, called intraluminal vesicles (ILVs), which bud from the internal lumen, giving a multivesicular appearance to the late endosomes (also known as multivesicular bodies, MVBs). From here on, late endosomes can take two different paths resulting in the degradation of their content (via endosome–lysosome fusion) or the secretion of the ILVs in the extracellular milieu through the fusion with the plasma membrane. These secreted vesicles are called EXOs [33]. ILV (EXOs precursor) biogenesis involves two main steps: (i) the formation of tetraspanin-enriched microdomains (TEMs) [34,35]; and (ii) the recruitment of specialized groups of protein complexes referred to as endosomal sorting complexes required for transport (ESCRTs) [36,37,38]. In the first step, tetraspanins are organized in highly concentrated domains prone to invagination. Tetraspanins are a conserved class of transmembrane proteins that act as scaffolding proteins, recruiting several molecules to a single area of a membrane, thanks to specific protein–protein interactions. Thus, TEMs form a network between themselves and surrounding molecules required for ILV formation. The complete maturation and budding of ILVs requires ESCRT machinery (zoom in Figure 2B), which includes ESCRT-0, I, II, III, and some accessory proteins. The ESCRT-0 complex binds and clusters ubiquitin-tagged proteins to be sorted into the vesicles [39]. The presence of ubiquitinated proteins and the curved membrane morphology trigger ESCRT-I and ESCRT-II recruitment [40,41]. The ESCRT-I complex is needed for membrane remodeling and the recruitment of ESCRT-III via programmed cell death 6-interacting protein (also known as ALIX). ALIX is an accessory protein that simultaneously connects a component of the ESCRT-I complex (TSG101) with a component of ESCRT-III (CHMP4) [42]. Finally, the ESCRT-III complex forms filaments that wrap the site of membrane constriction, assisting membrane budding and preventing cargo molecules from escaping into the cytosol [43]. EXOs contain several types of molecules, including proteins, lipids, and nucleic acids, but how the cargo is sorted into the vesicles remains unclear. ALIX and TSG01 are typical EXO markers together with tetraspanins such as CD9, CD63, and CD81. Despite the identification of various potential biomarkers for EXOs, their isolation is still a hard challenge. Evidence of an ESCRT-independent pathway of EXOs biogenesis suggests the presence of EXOs in which proteins such as ALIX or TSG01 could be absent [40,44]. Furthermore, tetraspanins are involved in several biological processes; therefore, they are not exclusive markers for EXOs [45].

### 2.2. Microvesicles

Unlike EXOs, which bud from intracellular membranes, MVs are generated straight from the blebbing of the plasma membrane. Their formation is a result of sequential changes in the enzymatic activity and the composition and morphology of the plasma membrane (Figure 2C). The latter is actively preserved in a state of asymmetry in terms of phospholipid composition, namely, phosphatidylcholine (PC) prevailing in the outer membrane leaflet, whereas phosphatidylethanolamine (PE) and phosphatidylserine (PS) prevail in the inner one [46]. The loss of asymmetry is a fundamental step required for the formation of MVs and depends on the activity of the lipid translocases (i.e., flippase, floppase, and scramblase), calcium-dependent transmembrane proteins that transfer lipids from one side of the membrane to the other. Flippase transports PE and PS from the outer to the inner side, while floppase transports PC outward [47,48]. Differently, scramblase is a bidirectional non-specific translocase, which flips the lipids randomly [49,50]. An increasing intracellular calcium level stimulates floppase and scramblase and turns off flippase, favoring a random distribution of membrane phospholipids. This lipid shuffling impairs the interactions with the underlying cytoskeletal components, leading to the loss of membrane–cytoskeletal anchorage and the subsequent formation of membrane regions prone to form blebs [51]. The last step prior to MV budding is the scission from the plasma membrane, which can involve the ESCRT machinery, as for EXOs, or occur via an ESCRT-independent pathway through the activation of the ADP-ribosylation factor 6 (ARF6) [52,53]. ARF6 starts a signaling cascade that culminates with the activation of the ERK pathway and the following phosphorylation of the myosin light chain. These phenomena trigger the contraction of the actomyosin network right under the bleb and ease MV release from the plasma membrane. MV content may reflect the antigenic state of the cell of origin, including a broad range of different molecules (e.g., enzymes, signaling proteins, mRNAs, miRNAs growth factors, and cytokines) [54]. MVs are characterized using flow cytometry for the presence of cell-specific surface markers together with PS [54].

### 2.3. Apoptotic Bodies

Apoptosis is a type of programmed cell death aimed to preserve tissue homeostasis and avoid aberrant cell replication [55]. This process is tightly regulated and can be triggered by cellular stress, infection, or DNA damage [56,57]. During apoptosis, a cell undergoes several modifications, including disruption of the cytoskeleton, chromatin condensation, nuclear fragmentation, and membrane blebbing [58,59,60]. The cellular content is disintegrated and the plasma membrane blebs vesicles of different sizes, the ABs, which contain cell debris, organelles, and nuclear material (Figure 2D). Clearance of apoptotic cells or ABs is operated by professional phagocytes or by neighboring cells. The formation of ABs facilitates the clearance with respect to a large, damaged cell. Especially, ABs are quickly and efficiently phagocytosed by surrounding cells, thus likely preventing secondary necrosis from occurring. Systematic changes in the AB’s membrane composition lead to the interaction with phagocyte receptors [61,62]. For a concise and informative description of the mechanisms behind AB clearance, we refer the reader to the recent excellent review from Battistelli and Falcieri [63]. As with MVs, the perturbation of the lipid membrane composition is a fundamental step in AB formation. PS is translocated outward of the membrane and interacts strongly and specifically with annexin V [64]. Furthermore, the oxidation occurring during apoptosis produces sites for the binding of the complement protein C3b or thrombospondin [65]. Annexin V, C3b, and thrombospondin are recognized by macrophage receptors triggering the phagocytosis. These proteins, as well as nuclear contents (i.e., histones and DNA fragments), are considered reliable markers for Abs [17].

## 3. Exosome Isolation

**Figure 3:**
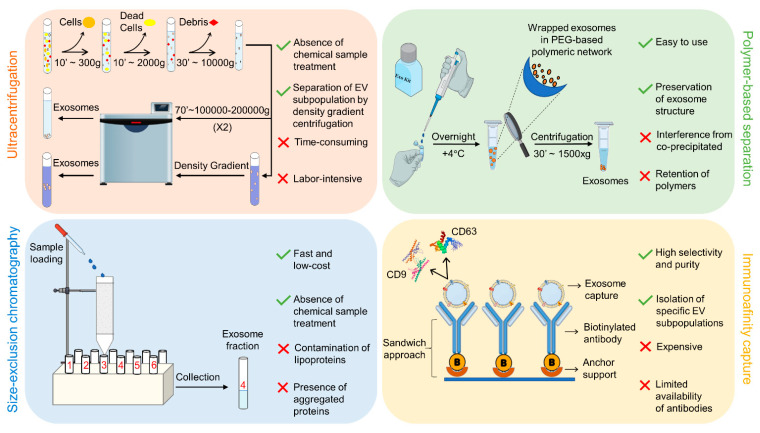
Schematic view of the different EXO purification methods.

As discussed in the previous sections, exosomes (EXOs) and extracellular vesicles (EVs) are heterogeneous in size, content, function, and origin [66,67], which makes isolation and purification a challenging task. For instance, some of the current isolation technologies are unable to completely separate EXOs from lipoproteins with similar biophysical characteristics and from EVs derived from non-endosomal pathways, resulting in low EXO purity [24,68]. To date, several techniques have been used for the isolation of EXOs that differ from each other in the physical, chemical, and biological principles exploited for separation from the biological matrix of origin (Figure 3). Therefore, the choice of separation and concentration method must be selected based on the experiment performed, and the most commonly used techniques are described below.

### 3.1. Ultracentrifugation

Ultracentrifugation (UC) is the most commonly used technique, and it is considered the “gold standard” for EXO extraction and separation. Thanks to the applied centrifugal force, the suspended particles are sequentially separated according to their physical properties and the physical properties of the solvent. Ultracentrifugation, also known as differential ultracentrifugation, mainly consists of two steps: first, a series of continuous low–medium speed centrifugation steps are used to remove dead cells, cell debris, and large-size EVs, and then high-speed centrifugation (at least 100,000× *g*) is utilized to separate EXOs. To inhibit the co-purification of lipoproteins [69] and soluble proteins [70,71], density gradient centrifugation (dg-UC) allows obtaining EXOs in a specific range of sizes compared with whole EXOs isolated by differential centrifugation [72]. Dg-UC is based on the ultracentrifugation of samples together with a nontoxic density-gradient medium of sucrose or iodoxinol [67,73,74,75]. As a general comment, UC is a conventional method suitable to separate EXOs from lipoproteins, EV protein complexes, aggregates, and other contaminants, but it has some drawbacks which hinder its use in clinical practice; it requires large sample volumes and expensive equipment, and it is time-consuming and labor-intensive.

### 3.2. Polymer-Based Separation

A hydrophilic polymer such as polyethylene glycol (PEG) is exploited to reduce EXO hydration, causing their precipitation as a consequence of an alteration in solubility/dispensability. Briefly, samples are incubated overnight with PEG precipitation solution (MW 8000 Da) [76], EXOs are wrapped in PEG, and then, after incubation, the precipitate containing EXOs is isolated using either low-speed centrifugation (1500× *g*) or filtration [77]. Currently, several commercial kits, such as ExoQuick, Exo-Spin, and Pure-Exo, exploit this mechanism, with some of them also being compatible with body fluids including serum, plasma, ascites, urine, cerebrospinal fluid, and culture medium [78]. ExoQuick is the most commonly used kit to isolate EXOs from various biomatrix because of its high purity and yield, as confirmed by proteomic and RNA profiling [73,79]. Although polymer-based methods are highly efficient, preserve vesicle structure, and are relatively easy to use, interference from protein coprecipitation is inevitable due to polymer/protein non-specific interactions. Notably, this issue can be resolved by combining different separation methods [80].

### 3.3. Size Exclusion Chromatography

Size exclusion chromatography (SEC) separates molecules that differ in sizes (hydrodynamic radius), and is widely used for the separation of biomolecules and chemical compounds, including proteins, enzymes, and antibodies [81]. This method was proven to be suitable for separating EXOs from several biological fluids, such as blood, plasma, urinary protein complexes, and lipoproteins [82,83,84,85,86] The stationary phase of the chromatography column can be packed with several gel polymers, including crosslinked dextrans (Sephadex), agarose (Sepharose), polyacrylamide (Biogel P), or allyldextran (Sephacryl) [87]. Commercially available EXO purification columns such as qEV separation columns, EVSecond purification columns, and Exo-spin are all based on the SEC principle. Although SEC is suitable for isolating EXOs in a uniform and narrow size range, leaving their biological characteristics unaltered, the presence of other particles with similar sizes leads to a reduced purity [88]. As a general comment, the disadvantages of this approach include the amount of work required, particularly when used in conjunction with other techniques, possible contamination of the sample with lipoproteins, and possible protein aggregation.

### 3.4. Immunoaffinity Techniques

EXO immunocapture allows for the separation of specific EXOs based on the expression of surface proteins. These proteins, including CD63, CD81, CD82, CD9, Alix, annexin, EpCAM, and Rab5, are specifically located on the EXO surface [89], contributing to the isolation of high-purity and specific subpopulations of EXOs. Antibodies (Abs) against these surface markers could be immobilized on a variety of media, including magnetic beads [90], chromatography matrices [91], plates [92], and microfluidic devices [93,94,95] for EXO capture. Each approach exploits the same principle of sandwich capture, in which the immunoaffinity media are functionalized with anti-Abs (Abs are CD63, CD81, etc.) and the EXOs are captured by the chemobiological interactions with their protein surface Abs. Among all media, magnetic beads have demonstrated a broad diagnostic and therapeutic potential [88,96]. Techniques based on immunoaffinity capture have a certain advantage, in particular, in obtaining EXOs with higher purity [97] than EXOs obtained by other methods, although commercially available antibodies are limited and very expensive, thus discouraging the use of this technique.

## 4. Scattering and Diffraction Provide Unique Information on EV Lipid Bilayer Arrangement, Composition, and Interaction with Nanosized Objects

Small-angle scattering (SAS) of X-rays (SAXS) and neutrons (SANS), wide-angle X-rays scattering (WAXS), and diffraction are techniques commonly used for the structural characterization of biological objects in a broad size range, from individual molecules and large complexes [98,99,100,101,102] to different tissue types [103,104,105,106,107].

SAXS and SANS are highly versatile techniques that can be used to retrieve the low-resolution shape of macromolecules in solution together with compositional information derived mainly from the tunable neutron contrast [98,108,109]. At variance with diffraction techniques, which require at least some degree of crystallinity, SAS can be applied to non-crystalline samples, a characteristic that makes it attractive for studying exosomes (EXOs).

**Figure 4:**
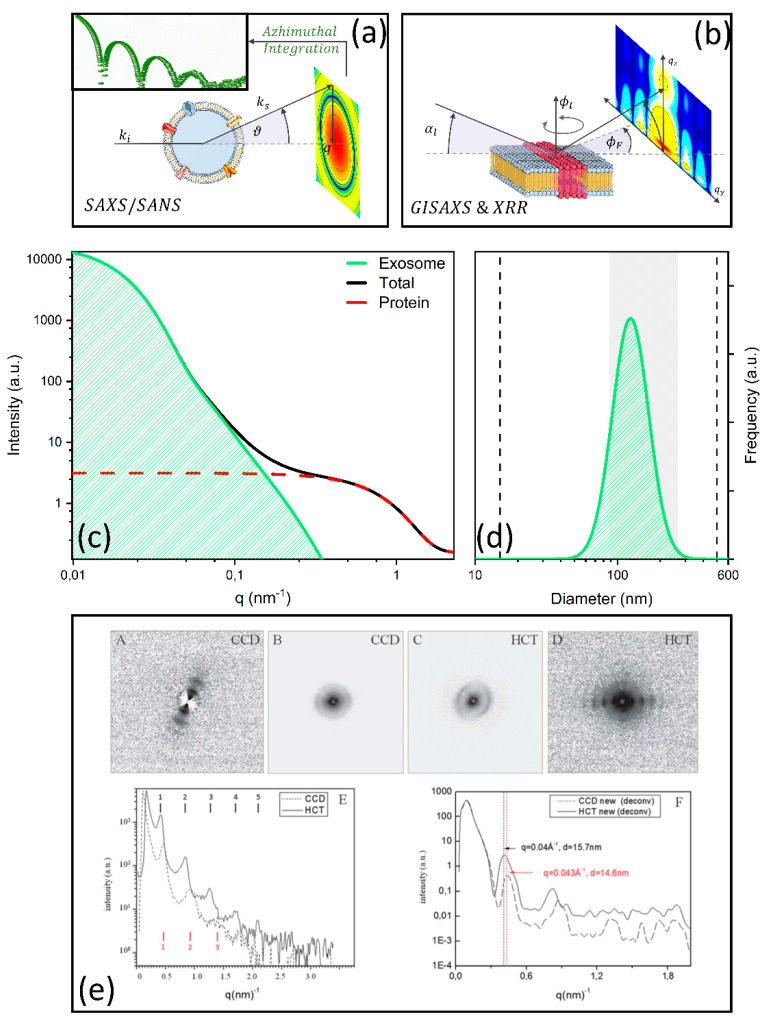
Schematic view of an SAS (**a**) and GISAXS experiment (**b**); simulation of the different contributions to the SAXS pattern measured by Varga et al. [121] on erythrocyte-derived EVs (**c**) with the corresponding distribution function (**d**). SAXS and WAXS profiles measured in [122] on exosomes obtained from healthy and cancer cells, pointing out the potential role of these techniques in exosome-based liquid biopsy. Data are reprinted with permission from ref [122] Copyright Royal Chemical Society, 2013 (**e**).

SAXS and SANS rely on the study of the angular dispersion—expressed in terms of the momentum transfer, q—of the scattered intensity, I (Figure 4a,b). In the case of monodisperse systems, scattering profiles (I versus q) can be studied with theoretical models based on the use of structure and form factors; for polydisperse and multicomponent systems, these factors need to be combined with size distribution functions [108], accounting for the variability of the relevant parameters. Alternatively, SAS profiles can be interpreted by comparing experimental data to numerical simulations, including—to mention a few—simulated annealing, statistical simulations, and molecular dynamic simulations [99,110,111,112,113,114,115,116,117].

SAXS profiles provide structural information at different scale lengths, from few angstroms to a few hundred nanometers, depending on the energy of the incident/scattered beam and the available q range. Thus, for particles such as EXOs (30–150 nm in diameter), SAXS is perfectly suited to retrieve EXO size, which can be obtained from the analysis of the Guinier/Porod region, EXO shape, as measured with form factors, interactions, as measured with the structure factor, and size dispersion. These analyses are not limited to EXOs, but can be performed for other vesicles [118], including liposomes [119] and synaptic vesicles [120].

The first SAXS application to extracellular vesicles (EVs) was demonstrated by Varga and collaborators, who exploited erythrocyte-derived particles [121]. EVs are extremely polydisperse by nature, and purified samples often co-precipitate with protein contaminants. The authors exploited SAXS to determine the diameter distribution function of the purified vesicles. For this purpose, three contributions to the scattering intensity were identified, namely, the EXO contribution I_EV_(q), the protein contribution I_P_(q), and background scattering I_BG_ (Table 2). The first two contributions were modelled using a core–shell and a spherical form factor, respectively.

Diameter dispersity was taken into account by using a log-normal distribution function. In Table 2, we summarize the full model exploited by the authors. In Figure 4c, we simulate the different contributions to the scattering pattern according to the fitting parameters reported in the paper. An analysis of the figure shows that SAXS allowed the authors to easily distinguish EVs from proteins, thus helping to remove contaminants in the computation of the EV diameter distribution. In Figure 4d, the SAXS distribution is reported together with the diameter range spanned by the distribution measured with dynamic light scattering (DLS) on the same sample (vertical black dashed lines). SAXS distribution appears to be significantly narrower than the DLS distribution. The authors further purified the measured sample using size-exclusion chromatography (SEC) coupled to DLS (SEC-DLS), to physically remove contaminants and measure contaminant-free EV diameter distribution. Very interestingly, the SAXS distribution spans a similar diameter range compared to SEC-DLS (grey shaded region in Figure 4d). Despite some limitations concerning the amount of sample required and the need for expensive instrumentation, the authors demonstrated that SAXS has the potential to serve as a reliable method for the traceable size determination of EXOs in solution, helping to remove contaminants through proper theoretical modelling of the scattering contributions.

Apart from size distribution, SAS can be applied to the determination of the EXO internal structure, with emphasis on lipid bilayer organization. Romancino et al. [123] combined SAXS and SANS experiments to elucidate the functional role of S-palmitoylation in the biogenesis of EVs secreted by skeletal muscle cells (C2C12 myotubes). S-Palmitoylation is a common lipid post-translational modification (PMT) in the human proteome and consists of the attachment of a saturated fatty acid—palmitic acid—to specific cysteine residues. This PMT enhances protein hydrophobicity and contributes to regulating biological processes such as localization, conformation stability, and protein–protein interactions at the membrane side. Interestingly, palmitoylated proteins are strongly enriched in EXOs compared to parental cells and MVs. This is probably associated with the fact that tetraspanins, which play a key role in the formation of endosomal sorting complexes (Section 2, Table 1), undergo palmitoylation to exploit their functions. Similar considerations can be assumed for the protein Alix, which was deeply studied in this paper, mainly using biochemical techniques. To study the effect of S-palmitoylation on the EXO lipid membrane structure, the authors studied skeletal muscle cells (C2C12 myotubes at the third day of differentiation) either untreated or treated with 2-bromopalmitate, which inhibits S-palmitoylation by interfering with the acylation/deacylation protein machinery. SAXS spectra of EXOs show a hump in the scattering intensity, centered approximately at q = 1.2 nm^−1^. This feature provides structural information at length scales of 2π/q = 5.2 nm in real space, and thus can be associated with the lipid membrane structure and arrangement in terms of phospholipid head-groups, hydrophilic tails, and transmembrane proteins. Interestingly, this spectral feature appears to be qualitatively different when comparing EXOs obtained from treated and untreated cells, showing that S-palmitoylation induces detectable changes in the overall arrangement and composition of the EXO membranes. To explore these structural differences in more depth, the authors exploited the information arising from neutron contrast variation. They showed that the hump in the scattering intensity was not observed in fully deuterated samples. In these experimental conditions, the contrast between phospholipid head groups and hydrophilic tails in the lipid membrane diminished significantly; thus, the authors hypothesized that the alteration in the measured hump could be ascribed to a structural change in the EXO lipid bilayer associated with the S-palmitoylation state. The authors limited their analysis to model-free observations, but indicated, as a possible theoretical framework, the use of onion-shell form factors. Taken together, these results show that SAS not only provides structural information on EXOs in a label-free fashion, but also gives detailed compositional information, especially regarding the EXO lipid content.

Study of the EXO external bilayer, their interaction with other surfaces and nanoparticles, as well as their structure, can be also performed using another kind of X-ray scattering technique, the so-called GISAXS, grazing incidence small-angle X-ray scattering (Figure 4b). This technique, which was specifically developed to study surfaces, combines features from SAXS and X-ray reflectometry (XRR). GISAXS has recently been employed in combination with XRR to study structural and adhesion proprieties of supported lipid bilayers obtained from extracellular vesicles (EVSLB), to develop synthetic surfaces that functionally and structurally resemble biological membranes [124]. In particular, the authors investigated the interaction between SLBs and superparamagnetic gold-coated iron oxide nanoparticles (SPIONs), which are a widely studied class of nanostructures with vast applications in hyperthermia, controlled release, and magnetic resonance imaging. For this purpose, the differences between EVSLBs and POCP-based synthetic support lipid bilayers (SLBs) were also evaluated. The authors showed that the GISAXS images of both membranes incubated with SPIONs displayed a signature at $q_{y}=0.17{Å}^{-1}$, derived from the oscillation of the specific form factor of nanoparticles. Interestingly such oscillation appeared to be much more defined on EVSLBs than on POCP-SLB. An analysis of the shape and the intensity of this feature allowed the authors to conclude that SPIONs are simply absorbed on the SLB surface, without membrane/nanoparticle reorganization, and thus without altering the membrane biomechanical response. A higher absorption degree was observed on the EVSLBs compared to POCP-SLB. A more in-depth model of the membranes was obtained using XRR. XRR curves were modelled as multilayers composed of a layer for the inner polar headgroup, a layer for the lipid chain, and a layer for the outer polar headgroup, each characterized by its thickness, scattering length density, and roughness. A further layer was added to account for SPIONs in the incubated samples. In agreement with AFM and QCM-D measurements, this analysis highlights higher roughness of the EVSLB compared to POCP-SLBs, associated with the protein content of EXOs, which is likely to contribute to the higher SPION absorption on the EV surface. This paper demonstrates that XRR and GISAXS can provide detailed and label-free characterization of the EXO membrane, providing in-depth information on the lipid bilayer structure and its interaction with other nanosized objects (results and methods are summarized in Table 2).

SAXS and WAXS were also applied for classifying EXOs obtained from healthy and cancer cells [122]. In this study, EXOs extracted from two different colon cell lines, CCD841-CoN (healthy epithelial colon cell line) and HCT116 (colorectal cancer cells), were investigated after drying on a nanostructured superhydrophobic PMMA surface. This surface was exploited to concentrate samples and to induce vesicle fusion that, in turn, leads to the formation of macroaggregates with lamellar structures. Micro-SAXS/WAXS was used to finely detect and quantify these features for diagnostic and classification purposes. Although both sample types showed similar microstructures under FIB-SEM imaging, SAXS/WAXS measurements highlighted a difference in the lamellar morphology, in terms of the number of orders, periodicity, and peak broadening. From the authors’ point of view, this is due to the more regular organization of the EXOs of HCT116 than those of the CCD841-CoN [122], which could be used to distinguish EXOs with different origins, also for diagnostic purposes. To stimulate the translational process of this technique, the authors also investigated the possibility to use a table-top instrument, instead of high-fluency synchrotron radiation sources. This was made possible thanks to a restoration algorithm that improved the visibility of diffraction peaks, beyond the first order, and consequently improved the accuracy in the lattice periodicity determination in the range of 0–1.8 nm^−1^ [125].

## 5. Vibrational Spectroscopies for Label-Free Exosome Molecular Profiling in the Omics Era

Vibrational spectroscopy (VS) techniques, such as Fourier-transform infrared (FTIR) and Raman spectroscopy, are emerging as major tools in contemporary diagnostics for the clinical evaluation of different types of human bioptic samples, including cells, tissues, biofluids, and extracellular vesicles (EVs) [25,126,127,128,129,130,131,132,133,134,135]. These methods primarily exploit the fact that chemical bonds within biomolecules absorb in the mid-infrared (IR) range of the electromagnetic spectrum, i.e., from 2.5 to 20 μm (4000 to 500 cm^−1^) as a consequence of the excitation of fundamental vibrational and rotational modes. These vibrational spectroscopies are also relatively easy to use, provide reproducible results, are largely non-destructive, and require relatively small amounts of material with little or no pre-processing steps. At a molecular level, these techniques allow direct access to the specific biomolecular absorption bands of proteins, lipids, and genetic materials which are found in exosomes (EXOs). The quantitative nature of spectral data provides further advantages over the conventional methods for the biochemical characterization of EXOs, such as ELISA, that often rely on the quantification of a single antigen/molecular type and require extensive labelling. In contrast to most conventional characterization methods, IR and Raman spectra can also be analyzed in an automated fashion, using multivariate statistical methods and machine learning approaches [131,135,136], which have the potential to provide physicians with direct diagnoses. These techniques also show some advantages if compared with conventional omics techniques, such as proteomics and lipidomics, giving complementary information. Although omics approaches provide more semiquantitative details on the specific molecular classes within EXOs than bulk vibrational spectroscopies, they involve complex sampling that can change the ratios of species. On the contrary, vibrational spectroscopies are perfectly suited to provide semiquantitative information on the relative amount of lipids, proteins, DNA, RNA, and carbohydrates in EXOs, also highlighting possible biochemical changes that depend on the clinical conditions of patients [137]. Moreover, vibrational spectroscopies are sensitive to biomolecules’ conformation, information which is not readily accessible to other techniques and that might be a potential source of clinically valuable information.

In the last decade, an increasing number of papers dealing with the spectroscopic characterization of EXOs have been published. Therefore, for the sake of clarity, we chose to summarize IR (Table 3) and Raman (Table 4) results separately.

### 5.1. FTIR Is an Effective Tool for the Label-Free Characterization of Exosomes and Allows for Their Automated Classification in Diagnostics

To the best of our knowledge, in 2015, Baddela et al. published the first FTIR application on EXOs [132]. Measures were acquired between 600 and 3600 cm^−1^ in the attenuated total reflection (ATR) mode. In this study, the authors characterized EVs isolated from buffalo’s milk. A commercial kit (Exoquick) for EXO extraction was used. Before IR measurements, EVs were characterized using dynamic light scattering (DLS), nanoparticle tracking analysis (NTA), and electron microscopy, showing a diameter distribution in the range of 50–200 nm. In all measured samples, IR spectra displayed peculiar absorption bands reflecting the EXO composition in terms of proteins, lipids, and genetic materials. More specifically, relevant absorptions were measured in the amide I–II regions (1500–1700 cm^−1^), C–H stretching (2700–3500 cm^−1^), and phosphodiester groups, phospholipids of nucleic acids, and C–O absorption of carbohydrates (900–1200 cm^−1^). A further comparison between spectral data and immune miRNA profiles was performed. Together, the results discussed in the paper show that the combined use of IR spectra and miRNA expression profiles can provide an efficient means to detect, quantify, and characterize bioactive compounds in buffalo milk, opening novel opportunities in food science.

**Figure 5:**
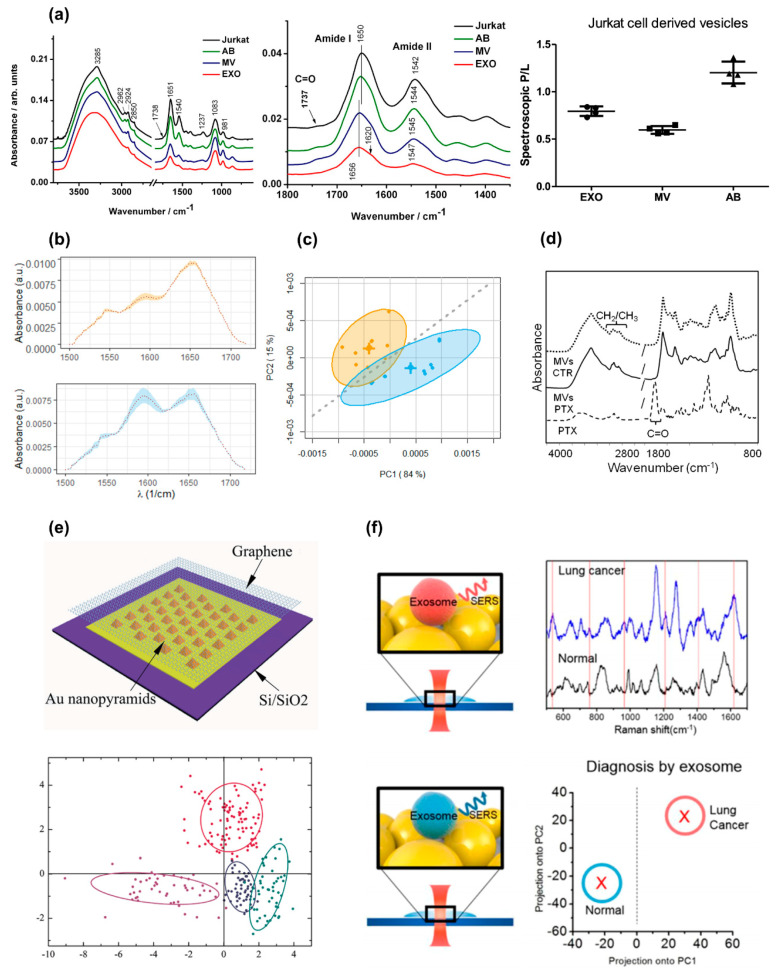
(**a**) Mid-IR characterization of EXOs MVs and Abs extracted from Jurkat T cells in ref [25]. Reprinted with permission from [25]. Copyright (2017) Elsevier. (**b**) Amide I and II bands measured on EXOs extracted from HT29 cancer cell lines well-fed and under serum starvation together with (**c**) the results of a PCA–LDA analysis [139]. (**d**) Mid-IR spectra of PTX-loaded molecules for drug-delivery purposes. Reprinted with permission from [134]. Copyright (2014) Elsevier. (**e**) Plasmonic nanopyramids for SERS of exosomes originating from different cell types together with the results of a PCA-based classification. Reprinted with permission from [141]. Copyright (2019) American Chemical Society. (**f**) SERS characterization of exosomes derived from human lung carcinoma (H1299, H522) and PAEpiC cell lines, with the corresponding PCA. Reprinted with permission from [142]. Copyright (2017) American Chemical Society.

A well-known issue in EXO research is the coexistence of diverse EV subpopulations with different relative concentrations in the purified samples. Such a wide sample heterogeneity has a detrimental effect on experimental reproducibility and data interpretation. Therefore, a simple and effective way for characterizing EXO samples, also capable of distinguishing different EV types, is highly demanded. Mihály et al. [25] used FTIR-ATR to tackle this key issue. For this purpose, EVs were isolated from Jurkat T cells, separating EXOs, MVs, and ABs using ultracentrifugation. The authors acquired mid-IR spectra of the three classes of EVs and the parental cells for classification purposes (Figure 5a). Interestingly, AB spectra strongly resembled those on parental cells. Subtle but detectable spectral changes were observed in the measured spectra, especially in the range 1800–1350 cm^−1^ that encloses the amide I and II bands. These changes included a slight shift in the amide I peak, which is centered at 1650 cm^−1^ for ABs and Jurkat cells, and 1656 cm^−1^ for AB and EXOs (Figure 5a). This modification hints at a variation of the secondary structure content among the different types of samples. Moreover, the relative weight of the amide I and II peaks appears to decrease with the average EV size (ABs > MVs > EXOs) compared to a spectral component at approximately 1600 cm^−1^, which is attributed to protein aggregation and amino acids through spectral deconvolution. The authors also highlighted a promising mid-IR quantitative maker capable of discriminating among the three EV subtypes. This marker is referred to as the protein–lipid spectral ratio (P/L) and is computed as the ratio between the integrated intensity of the amide I–II absorption band (1750–1500 cm^−1^) and the lipid CH stretching band (3040–2700 cm^−1^). Specifically, MVs possess a larger P/L than EXOs which, in turn, show larger values than ABs (Figure 5a). Interesting, only for ABs was the P/L value greater than 1, similarly to what can be measured on Jurkat cells.

Several recent papers in the literature investigated EVs biochemical modifications in vitro due to different cellular treatments, e.g., controlled variations in the cellular medium.

In this context, Lee and co-workers used FTIR-ATR to detect and quantify subtle biochemical changes in MVs released from monocytes (THP-1 cells) upon lipopolysaccharide stimulation (LPS) [137]. This investigation is particularly relevant if one takes into account that monocytic-derived MVs are likely to play an active role in immune responses, as a consequence of altered lipid content and increased levels of RNA and proteins that, in turn, can actively affect the target cell biochemistry. The authors succeeded in demonstrating that monocyte activation can be inferred from the analysis of released MVs. This was made possible through a careful comparison between mid-IR spectra of cells and MVs extracted from these cells, before and after LPS. This comparison showed that spectral changes in MVs upon LPS mimics spectral changes in the parental cells. In the comparison, an analysis of the integrated areas of the lipid ester, α-helical protein, and uracil bands showed a significant increase upon LPS stimulation. Similar changes were detected on monocytes upon LPS stimulation. The similarity of the spectral changes was also confirmed by an analysis of the PCA loadings. Taken together, these results show that FTIR spectra from MVs can provide novel biochemical insights into the LPS-induced monocyte model of septic shock. Moreover, this study also demonstrates that a mid-IR analysis of MVs can be directly related to changes in the cellular phenotype.

Pereira et al. revealed the influence of culture and time conditioning in EXOs released from human bone marrow mesenchymal stem/stromal cells (BM-MSCs) [138]. For this purpose, BM-MSCs from six donors were cultured in two different media: (i) conventional DMEM and (ii) Stem Pro^®^ MSC SFM XenoFree medium. A comprehensive analysis based on the use of PCA, first and second derivatives highlighted those factors affecting most of the biochemical composition of EXOs. It was shown that the IR signatures were more significantly dependent on the medium than on the MSC donor or the conditioning days. These results highlight the key role of the different culture conditions in EXO research, emphasizing that great attention must be paid to this particular aspect to assure experimental reproducibility.

Similarly, Romanò et al. used FTIR spectroscopy in the mid-infrared (mid-IR) range to detect biochemical differences in EXOs released from human colorectal HT-29 cancer cells in different culture conditions [139]. Cells were grown both in well-fed conditions and under serum starvation. Data showed the presence of statistically significant differences in the shape of the amide I and II bands in two conditions. The authors showed that these subtle differences in the spectral shape of the amide absorption bands could be used to automatically classify EXOs extracted from the two types of cells using PCA combined with linear discriminant analysis (LDA). Interestingly, testing the classifier performance, the authors obtained very high accuracy, precision, and recall, especially in the amide I–II regions (Figure 5b,c). These results confirm that FTIR spectroscopy on cell-derived EXOs is a useful tool to gather information on the cellular state.

In 2014, Pascucci et al. first applied FTIR spectroscopy to the characterization of MVs loaded with anticancer molecules for drug-delivery purposes [134]. The authors exploited an interesting capability of mesenchymal stromal cells derived from bone marrow: upon exposure to high Paclitaxel (PTX) concentrations, they first incorporate PTX and then release it within MVs. PTX incorporation was assessed with HPLC before IR measurements. It was shown that drug loading induced a significant change in the MV spectral profiles between 3000 and 2800 cm^−1^, i.e., where CH stretching modes occur (Figure 5c). Enlarged details of this absorption band showed the presence of new and specific features in MV spectra that corresponded to those of PTX in the same spectral region (Figure 5d). This interesting application demonstrates that the label-free characterization of EVs with vibrational spectroscopy can provide a quick and effective way of controlling EXO-based nanocages for drug delivery applications.

One of the most promising areas of EXO research is its possible use as a cancer biomarker in liquid biopsy and personalized medicine. This rapidly evolving field would greatly benefit from the development of fast and effective characterization methods using vibrational spectroscopies.

In this context, Krafft et al. collected MV-enriched and EXO-enriched EV samples from patients diagnosed with prostate cancer and non-cancer patients and healthy donors [133]. The authors suggested that a reduction in the alpha-helix secondary structure content and of beta-sheets content of the EXO enriched sample can be a cancer-specific blood EV marker.

Zlotorogski-Hurvitz et al. recently published one of the most articulated clinical applications of IR-based molecular profiling of EXOs. The study aimed to investigate the possible use of FTIR spectroscopy for the classification of EXOs extracted with ultracentrifugation from oral cancer patients and healthy individuals. This study is extremely interesting for many reasons, including the fact that data relied on a robust sample size, which comprised 21 patients diagnosed with oral cancer and 13 healthy subjects. Considering that FTIR is adjusting the first steps in EXO diagnostics, the number of enrolled subjects in this study is quite remarkable, thus providing—to the best of our knowledge—one of the first validations of this approach in a clinical setting. The authors highlighted a significant difference in IR spectra between the two groups at 1072 cm^−1^ (nucleic acids), 2924 cm^−1^ and 2854 cm^−1^ (membranous lipids), and 1543 cm^−1^ (transmembrane proteins). As often occurs, such a difference is highlighted through relative intensity ratios. Specifically, patients showed increased ratios compared to controls in the following cases: relative intensity ratio of 1033 cm^−1^ and 1072 cm^−1^ (I_1033_/I_1072_), I_2924_/I_2854,_ and I_1404_/I_2924_. PCA–LDA was used to build a model for subject classification, which showed a sensitivity of 100%, specificity of 89%, and accuracy of 95%. Further validation in a clinical setting was published by Yap et al. in 2019, comparing measurements on EXOs extracted from prostate cancer donor cells and five healthy individuals’ control cells [140]. IR spectra showed interesting differences in the wavelength range 1794–813 cm^−1^.

A further compelling clinical validation of an EXO-based liquid biopsy approach for the diagnosis of Alzheimer’s disease (AD) was recently published by Martin et al. [135]. In their study, a total of 21 AD patients and 21 healthy donors (HD) were recruited, which is a remarkably robust sample size at the present research stage in this field. For this purpose, recruitment was performed in two cohorts of subjects in the context of a multicentric study. The authors compared the FTIR spectra of serum and serum-derived EXOs in both groups. Serum and serum-derived EXO spectra were qualitatively similar, with some notable differences. Firstly, EXOs had higher absorbance than serum spectra in the lipidic regions (3000–2800 cm^–1^ and 1483–1423 cm^–1^). Secondly, EXOs had a higher absorbance in the 1200–900 cm^–1^ region, associated with the presence of nucleic acids and carbohydrates. In this range, an intense peak was observe at 1064 cm^–1^, assigned to symmetrical ester C–O–C stretching of phospholipids and/or ribose C–O stretching (nucleic acids). The authors performed an in-depth multivariate analysis in the latter region, using PCA applied to the second derivative spectra. Notably, PCA distinguishes the two cohorts of subjects, in such a way that samples derived from the different cohorts cannot undergo the same multivariate analysis. This is probably due to different serum collection/processing procedures, thus stressing the strong effect of these aspects in EXO research for diagnostic applications. As expected, PCA on serum-derived EXOs allowed for better discrimination of the two groups in both cohorts when compared with serum samples. PCA–LDA and PCA–QDA were also performed, obtaining similar results: serum-derived EXOs presented a higher discriminatory power compared to unprocessed serum. PCA loadings were investigated to highlight the peaks responsible for discriminations between the two groups. The selected peaks were further compared in the framework of univariate analysis. Very interestingly, a significant difference was found at 1064 cm^−1^ for both cohorts. Taken together, the results discussed in this study are extremely interesting from both a methodological and a clinical point of view, because a blood test capable of diagnosing AD is still lacking, which is a matter of intense research.

### 5.2. Exosome Characterization with Raman Spectroscopy: From Bulk Sample to Single Molecule

Raman spectroscopy and related inelastic scattering techniques represent an effective and versatile approach for the label-free characterization of EXOs. As a non-destructive technique that returns a chemical characterization of the samples, Raman spectroscopy is useful not only for the theoretical study of EVs, but also as a diagnostic tool for the early detection of cancer and other diseases. To the best of our knowledge, its first applications to EVs date back about ten years [143,144]. After that, we observe a rapid increase in the number of papers published in this field, also due to technological advancements such as surface-enhanced Raman spectroscopy (SERS) and Raman tweezers microspectroscopy (RTM).

One of the traditional drawbacks that limited the use of Raman spectroscopy is the weakness of the Raman scattering intensity. However, the use of specific nanostructured systems, such as patterned surfaces and plasmonic nanoparticles, can locally lead to a dramatic enhancement of the Raman signal, even by a factor of 10^9^ to 10^11^. This is the principle of SERS, which represents the most widely utilized approach to characterize the EXOs using Raman spectroscopy [145]. The first implementation of SERS for EXO characterization was reported by Tirinato et al., who obtained the spectra from human colon epithelial cells (CCD841-CoN) and human colorectal cancer cells (HCT-116) by using SERS combined with superhydrophobic surfaces (SHS) [146]. It is worth noting that SHS surfaces are widely used in different applications to concentrate and manipulate biological samples at a nanoscale level [122,146,147,148,149,150,151,152]. In the paper from Trinato et al., a silicon micropillar array created the SHS, concentrating the EXOs in a small area where silver nanograins enhanced the electromagnetic field, and consequently, the Raman signal. This setup allowed, for the first time, the recognition of some relevant differences in the Raman spectra obtained from EXOs extracted from normal and cancer cells. Henceforth, many other label-free SERS approaches have been exploited for EXO characterization. Most of them relied on the use of gold nanoparticles to create an SERS substrate [142,153,154,155], or to form a solution composed of EXOs aggregated to Au nanoparticles (GNPs) [156,157]. Other SERS techniques used for EXO characterization are based on different nanostructured arrays, such as high-density Au nanorod (NR) array substrates with Ag nanocubes (NCs) assembled on the NR hot ring [158], nano-bowl arrays covered by a thin Ag film [159], and a hybrid substrate consisting of a graphene-covered Au surface containing a quasi-periodic array of the pyramid [141] (Figure 5e). However, the use of SERS is still limited to the academic environment, and its clinical application is inhibited due to high costs and technical requirements for substrate fabrication. In this context, Avella-Oliver et al. proposed an interesting approach to reduce the cost of SERS substrates by using regular recordable disks covered with silver [160], and successfully tested their substrates on the EXOs extracted from a lung cancer cell line (A549 UC).

SERS is currently the most utilized approach to study EXOs with Raman spectroscopy, but is not the only one. Since 2012, optical tweezers have been combined with Raman microscopy to disclose the composition from a few to a single EXO [161]. SERS indeed has some critical drawbacks which limit its potentials. Firstly, the enhancement of the Raman signal sharply diminishes with the distance from the functionalized SERS surface or nanoparticles, annihilating them in a few nanometers. For this reason, most of the signal captured by the Raman detector originates from the components of the EXOs which are closer to the SERS substrate, primarily the membrane and the molecules in its proximity. RTM, in contrast, prevents the underestimation of contributions from the molecules inside the EXO, because the EXO is entirely within the optical trap and the signals come from the whole vesicle. This, combined with the possibility to acquire the signal from a few to even a single EXO, enabled researchers to determine a reliable fingerprint for the EXOs and also distinguish some subpopulations within the EXOs derived from the same cells [162,164]. Without the effect of the substrate, which enhances the Raman intensity, other strategies must be employed to improve the signal quality. These ranged from air-drying the EXOs [163] (even without using an optical tweezer) and removing noise from the solution, to the optimization of acquisition setup and protocols [164]. Nevertheless, the intrinsic weakness of the RTM signal usually leads to the lengthening of the acquisition time as a drawback.

Most of the recent interest around EXOs has been driven by their promising usage as a cancer biomarker, through their isolation and analysis from liquid biopsies. In this context, Raman was identified as a potential candidate to accomplish this task, allowing the detection of small differences in the sample composition. Thus, in the last decade, a large portion of the studies which involved Raman spectroscopy for EXO characterization focused on the possibility of distinguishing EXOs secreted from cancer and healthy cells, in the perspective of diagnostic applications. As mentioned above, the first steps toward the application of label-free Raman for cancer detection date back to 2012, with two pioneering studies from different research groups. Tirinato et al. applied SERS on SHS surfaces for the characterization of the spectra from Human colon epithelial (CCD841-CoN) and human colorectal cancer (HCT-116) cell lines [146], whereas Tatischeff et al. proved the applicability of RTM to detect changes induced by starvation on Dictyostelium discoideum cells by analyzing their EXOs [161], also reporting the first attempt to characterize EXOs extracted from the urine of human patients. The Raman capacity to reveal modifications in the EXO parental cell conditions was confirmed in 2014 by Kerr et al., which found relevant differences between the spectra of EXOs from ovarian carcinoma cells (A2780) grown in hypoxia and normoxia conditions [156]. This article also reported a useful comparison between gold nanoparticle SERS and Raman microspectroscopy, highlighting the necessity of fine control and optimization of the SERS parameters to avoid thermal damage.

Aside from these applications, Raman was also employed for the evaluation of the different extraction techniques. The standardization and validation of the extraction methods are still one of the most critical requirements for the usage of EXOs in clinical practice. The capacity of SERS to detect the changes in the molecular composition of the EXO membranes allowed Lee et al. to compare the purity of the EXOs obtained through differential/gradient ultracentrifugation with those from the commercial isolation kit [159]. Using an ovarian cancer cell line (SKOV-3) as a model and a thin silver film-coated nanobowl SERS, they demonstrated a relevant dependence between the EXO extraction techniques and the shape of the Raman spectra, with the products derived from the commercial kit which showed several peaks that could be associated with the presence of molecules from the isolation solution. These results suggested the use of differential/gradient ultracentrifugation methods as a gold standard for the extraction of high-purity EXOs.

In the following years, there has been much focus on the research of cancer signatures in Raman spectra obtained from EXOs. Smith et al. used Raman to characterize the spectra of the EXOs derived from both cancer and non-cancer cell lines [162]. In this seminal work, the research was expanded to a total of seven different cell lines: human lung carcinoma A549, human hepatocarcinoma Huh-7, human ovarian carcinoma SKOV3, human acute myeloblastic leukemia Kasumi-1, human acute T cell leukemia Jurkat, mouse embryonic fibroblast 3T3, and human lung normal fibroblast IMR90. Interestingly, because they used an experimental approach that allowed measuring all the spectra from a single EXO at once, they were able to find variances not only between cancer and normal cell lines, but also within the EXO from the same cell line. In particular, they identified four major subpopulations with different weights, shared among all the cell types, suggesting a specific biological role for each of them. On the other hand, the main difference between EXOs derived from normal and cancer cells appeared to be related to the relative expression of membrane lipids, with cancer cells which showed lower values of cholesterol and a higher content of phospholipids. Some other, more recent studies have followed this direction, and successfully tested Raman capacity to distinguish cancer EXOs on a large number of different cell types. Zhang et al. recently proved the SERS capacity to distinguish EXO from eight different cell lines: human esophageal cancer cells (EC109, EC9706, and Kyse150), cancerous breast epithelial cells (M231 and MCF7), hepatoma cells HepG2, human normal hepatocyte cells (L02) and human nontumorigenic breast epithelial cells (MCF-10A) [157]. Sivashanmugan et al. used human bronchial epithelial cells (NL-20 and Beas-2b), the murine lung fibroblast cell line (L929), and three different human lung adenocarcinoma cell lines (PC9, HCC827, and H1975), and found that the cancer cells released a more heterogeneous population of EXOs, with differences not only in membrane lipids, but also in the protein compositions [158]. In this regard, Shin et al. acquired the Raman spectra of non-small cell lung carcinoma (NSCLC, PC9 and H1299) and human pulmonary alveolar epithelial cells (HPAECs), and correlated the changes observed with the spectra of some specific protein markers [154]. This approach demonstrated a strong association between several markers and some of the peaks observed in NSCLC Raman bands. The difference between NSCLC and pulmonary alveolar epithelial cells (ScienCell) EXOs was previously studied by Park et al., in 2017 [142]. Using SERS, they demonstrated an excellent ability to distinguish the EXO origin from the Raman spectra. Interestingly, they also attempted a further step toward the application of SERS in the diagnostic field by testing their approach on EXO extracted from the serum of two healthy people and two patients with lung cancer, but the results showed that this technique was still not ready for clinical use. The problem especially addressed the high heterogeneity of the EXO population in a real blood sample, among which just a very low percentage originated from cancer cells. Stremersch et al. already proved the capability of SERS to distinguish different mixtures of RBC and B16F10 melanoma cell lines, successfully detecting and quantifying the presence of EXOs from B16F10, but in an artificial mixture with an enhanced percentage of EXO from a well-known cancer cell line [153]. As also proved by Yan et al. [141] EXOs extracted from a blood sample of a healthy human differ significantly, from a statistical point of view, from EXOs secreted by cancer cells (lung adenocarcinoma cell lines HCC827 and H1975), and interestingly, also from EXOs extracted from bovine serum.

Summarizing all these results, the main contribution to the changes between normal and cancer cells EXOs has been identified in their membrane constituents, namely, the principal membrane lipids and proteins. These results could be influenced by a technical bias induced by the use of SERS, which enhances signals from the molecules in the proximity of a functionalized SERS surface. However, Gualerzi et al. arrived at a similar conclusion by using Raman microspectroscopy, which can detect the membrane constituent of the EXOs as well as their bulk components. The enhancement of the signal was obtained by water evaporation, analyzing the air-dried drops of an EV suspension. In this study, they compared the signal of the EXOs isolated from human mesenchymal stromal cells (bone marrow mesenchymal stromal cells and adipose tissue mesenchymal stromal cells) and dermal fibroblasts, demonstrating the capacity of Raman microspectroscopy to distinguish vesicles from undifferentiated and differentiated cells. In order to return a more comprehensive picture of the biomolecular components of EXOs, from membrane to internal molecules, Kruglik et al. recently employed RTM for the analysis of EXOs isolated from rat hepatocytes (control and treated with the hepatotoxin) and from the urine of two healthy human donors. They demonstrated the RTM ability to reveal the presence of different nucleic acid contents for EXOs extracted from different and, surprisingly, even from the same samples. Enabling detection from a single to a few EXOs for each measurement, the application of RTM enabled identification of a strong intra-sample heterogeneity in the EXO biomolecular components, especially for the human-derived vesicles.

Just some of the aforementioned studies reported spectral data from human EXOs, directly extracted from processing human liquid biopsies. None of them were primarily focused on this topic, but they described a high heterogeneity in these data, hindering the transition to clinical applications. However, consistent statistics were still missing before Shin et al. published the first extended study on the application of Raman for the classification of human blood-derived EXOs [155]. Using SERS, they analyzed EXOs from 63 patients (20 healthy and 43 with lung cancer) and compared the spectra with EXOs obtained from normal and cancer cell lines (HPAEpiC, A549, H460, H1299, H1763, and PC9), developing a deep learning model which could distinguish healthy and cancer patients with high accuracy. These results encourage research on the application of Raman as a diagnostic tool for cancer early detection, even if the road for the transition from basic research to clinical application in cancer diagnosis is still very challenging.

As demonstrated by Shin et al., one of the crucial requirements for the application of Raman to EXO classification relies on the development of robust and effective algorithms of analysis. The interpretation of results related to the aforementioned studies is strictly related to the analysis method performed in the EXO RS research activities. In fact, in the first phase, a ratiometric approach, along with standard peak analysis, has been selected by researchers in order to qualitatively distinguish the spectral shape and identify the characteristic peaks associated with functional chemical groups.

In this context, multivariate analysis serves as an extremely powerful tool to separate diagnostic information from a statistically complex background. Principal component analysis, together with other multivariate analysis techniques such as LDA and DFA, appeared to be some of the most frequently applied tools recently employed in the application of RS to the EXO field, as found, for example, in [142,154,157,159].

The ultimate step of data analysis, which is now gaining interest, is currently represented by the most up-to-date machine learning (ML) tools, such as (auto)trained algorithms based on the previously cited techniques or MCR–ALS rather than PLS–DLA: the essential idea relies on the imitation of deep learning strategies to infer statistical interpretations of the available RS datasets. ROC curve analyses among all data handling techniques provide an efficient way to compare them all.

## 6. Nanoindentation: Searching for an Exosome Mechanical Fingerprint of the Disease

**Figure 6:**
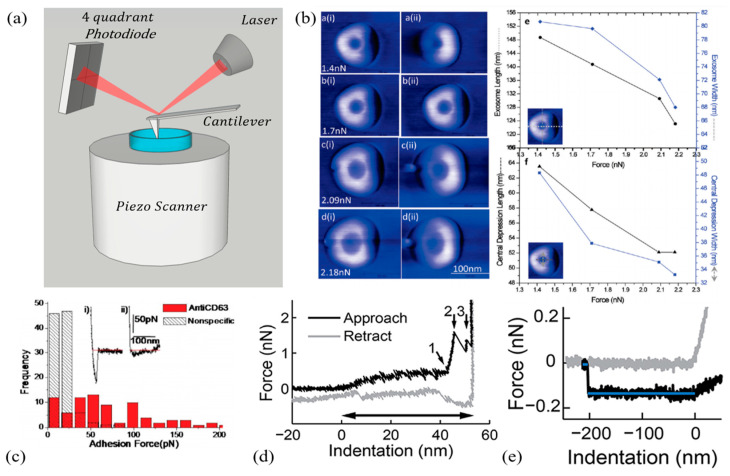
Schematic view of a typical AFM setup (**a**); dependence of the exosome shape on AFM applied force in the range 1.4–2.4 nN (**b**) and specific adhesion on exosome expression CD63 (**c**). Reprinted with permission from ref [190]. Copyright ACS (2010). A typical shape of a correct indentation curve on exosomes (**d**) together with the mechanical signature of EXO membrane tethering (**e**). Reprinted with permission from ref [174]. Copyright Frontiers (2020).

Atomic force microscopy is a well-known technique, which allows the acquisition of high-resolution topographical images of biological samples with a lateral resolution comparable to scanning electron microscopy and an unparalleled vertical resolution down to the sub-nanometer level. Additionally, experiments can be carried out under physiological conditions and without the need for extensive pre-processing steps, which might significantly alter the sample, introducing severe artefacts. Information is gathered by scanning an elastic cantilever with a nanometric tip on the sample surface [165,166,167]. Cantilever deflection is recorded thanks to an optical setup coupled with high-precision piezoelectric actuators (Figure 6a). Aside from imaging purposes, the same cantilever can be used as a sensitive mechanical probe to indent the sample, acquiring spatially resolved images of its mechanical properties [168,169,170,171,172,173,174]. This operation mode is often referred to as force spectroscopy, and it is remarkably flexible, allowing scientists to measure a wide range of mechanical parameters, such as stiffness, Young’s modulus, adhesion force, work adhesion, hysteresis, dissipation, and relaxation times [113,175,176,177,178,179,180]. As a further degree of freedom, different types of tips and cantilevers can be chosen [181], and custom relaxation curves can be designed to monitor the mechanical response of the sample over time [171,173,176,182]. Interestingly, AFM can be coupled to nanoscale IR spectroscopy (nano-IR), exploiting tip-enhanced plasmonic effects [183]. This highly versatile technique, albeit time-consuming, is extremely powerful because can obtain a wide variety of information, encompassing structural, mechanical, thermal, and biochemical properties. Notably, in contrast to most conventional microscopy techniques, all this information is quantitative and can be analyzed with advanced statistical methods in an automated fashion using machine-learning approaches [184,185,186,187]. All these characteristics make AFM a promising tool for the search and validation of novel label-free exosome (EXO)-based biomarkers for biomedical and diagnostics applications. In this regard, a caveat is necessary, because this exceptional versatility is both a blessing and a curse, often making comparisons among different papers a challenging task. A typical example is provided by Young’s modulus (E), which is affected by several parameters, including the scanning velocity during indentation, the indentation force, the tip shape, and the environmental condition [188,189].

In this review, we have limited our discussion to EXO mechanical characterization, while neglecting imaging applications. For a concise summary of EXO imaging, we indicate the excellent review from LeClaire and co-workers, which also carefully summarizes the three main mechanical models exploited in EXO experiments, namely, the Hertz/Sneddon, the Thin Shell, and the Canham–Helfrich models [191].

Currently, the AFM mechanical characterization of EXOs appears to be still in its infancy, with only a few papers published on the subject [174,190,192,193,194]. Among these papers, most of them cover mainly methodological aspects, also attempting to establish a shared methodology for EXO characterization, thus reducing the aforementioned variability (see Table 5).

Sharma et al. first studied the structural and mechanical features of EXOs extracted from saliva samples by using ultra-sensitive low force AFM in amplitude and phase modulation mode [190]. The authors investigated the structural changes of EXOs under varying forces in the range 1.4–2.4 nN, identifying progressive nanoparticle deformation accompanied by the formation of a tri-lobed-shaped depression region in the particle center. The larger the applied force, the deeper the depression was (Figure 6b). Such deformation appeared to be reversible under low forces. Conversely, under forces exciding a given threshold, EXOs underwent an irreversible rupture. The severe EXO deformation under applied force was a remarkably reproducible feature among different AFM studies. This characteristic points to the need to use forces as low as possible to minimize particle deformation and rupture (see Table 5). Additionally, the authors characterized the salivary EXOs using AFM in the spectroscopy mode to measure the specific adhesion between CD63 antigens on the EXO surface and a functionalized tip, showing the AFM potential to perform specific EXO detection and recognition at a single-molecule level (Figure 6c). This is a key result for EXO diagnostic applications because it provides a means for the experimenter to select EXOs expressing a particular antigen associated with a pathological state and to determine, on a statistical basis, the frequency of its occurrence in a sample population.

Li et al. used AFM in the peak force tapping (PFT) mode to simultaneously measure EXO topography and mechanical properties in terms of Young’s modulus E, deformation, adhesion, and dissipation [194]. For this purpose, the authors extracted EXOs from bone marrow biopsies of lymphoma patients. Measures were carried out in a liquid environment with EXOs dissolved in PBS and immobilized on poly-L-Lysine-coated glass slides. The authors showed that poly-L-Lysine facilitates AFM measurements of EXOs, which displayed a large height/diameter ratio (~0.3) compared to air-dried EXOs, which are more affected by artefacts. PFT was demonstrated to be suitable for the quantitative imaging of EXO Young’s modulus, highlighting the stiffness contrast with the substrate. Conversely, adhesion between the tip (silicon nitride) and EXOs was not significantly different from that between the tip and the substrate. Interestingly, EXOs did not show a homogeneous spatial distribution of energy dissipation. Together, the results of this paper demonstrate that AFM in the PFT mode is suitable for the quantitative analysis of EXOs; thus, it is of potential use for the search of EXO-based biomarkers.

Concerning AFM indentation-type experiments, Parisse et al. first reviewed the characteristics of a typical FD curve acquired on SKBR3 EXOs in a liquid environment [195]. This curve type can be measured with a conventional AFM setup in the force spectroscopy mode, and it is qualitatively in agreement with curves acquired on lipid vesicles deposited on a hard surface. This resemblance has led scientists to develop more comprehensive mechanical models of EXO biomechanics that could be validated using artificial vesicles whose size and composition can be finely tuned according to experimental needs. In this context, the recent work of Vorselen et al. is particularly relevant because it addresses the issue of establishing a reproducible protocol for the mechanical analysis of EXOs in a liquid environment, encompassing sample pre-processing protocols, measurements, data analysis, and possible limitations/problems [174]. The authors used poly-L-lysine to promote adhesion on the substrate because AFM mechanical measurements are negatively affected by loosely attached samples [196]. Unfortunately, EXOs’ adhesion comes along with a non-negligible particle deformation, which changes from a spherical shape to a cup shape. This is a common phenomenon for EXOs due to electrostatic interaction. This deformation pressurizes EXOs, and two main contributions have to be taken into account when dealing with their mechanical properties, namely, membrane bending modulus and La Place pressure, which is computed from the knowledge of the membrane surface tension and the particle radius. The greater the particle deformation, the larger the La Place pressure, which is often the dominant contribution. To decouple these contributions in the framework of the Canham–Helfrich model, authors combined the information coming from EXO indentation (Figure 6d) and membrane tethering (Figure 6e), which is measured from a plateau in the retract curve. Measuring membrane tethering might be a challenging task because a non-clean tip modifies the retract curve. In this regard, the authors suggest that the reader acquire an FD curve on the poly-L-lysine-coated glass to check for a clean tip. Very interestingly, the authors discuss the correct shape for an EXO FD curve, which should present the following hallmarks: (i) a smooth indentation between the contact point and the EXO height, excluding the bilayer thickness; (ii) an increase in the reaction force when the two bilayers are pressed together (arrow 1); (iii) two sharp discontinuities corresponding to the first (arrow 2) and second (arrow 3) bilayer rupture; and (iv) the contact between the cantilever and the substrate. This method allowed the authors to discriminate different types of EXOs in a clinically relevant context, showing mechanical differences between healthy donors and patients diagnosed with hereditary spherocytosis [192].

A key problem hindering the translational process of AFM biomechanics in clinical practice is the great amount of time necessary for data collection. This crucial issue has been faced in the paper by Ridolfi et al., who developed a novel methodology to perform mechanical characterizations of EXOs starting from an AFM-based contact angle measurement. This method is extremely interesting because can characterize hundreds of individual EXOs in less than one hour, thus paving the way for AFM high-throughput application in this field [193].

Taken together, the results summarized above show that AFM is a versatile and promising tool for the search and validation of novel EXO biomarkers of disease, but it is also plagued by severe limitations which are hindering its spread in diagnostics. These limitations include long experimental times, the need for specialized personnel with a strong mathematical background, and the inherently low statistics in AFM experiments. In this regard, we stress that a significant step to reduce experimental and analysis times has been achieved in the paper of Ridolfi and co-workers [193]. An intriguing approach to overcome the small sample size is exploited in ref [174], where a bootstrap method is used to estimate mechanical parameters with their confidence intervals. Except for a few papers [113,174,193,197], bootstrap techniques are rarely used in AFM experiments, but the AFM community would probably benefit from the use of advanced statistical methods that allow for the treatment of a small sample size. The need for more advanced statistical methods is even clearer if one considers that the AFM community is often poorly familiar with statistical figures that are widely used in clinical practice. To show the potentiality of this technique in diagnostics, researchers could consider adopting the same statistical language used by clinicians when presenting novel biomarkers. For instance, instead of just indicating the presence of statistically significant differences, a more convenient discussion could include ROC curves and survival curves when appropriate [170,198], two widely used statistical figures for assessing the diagnostic and prognostic performance of a biomarker, together with a discussion on biomarker specificity, sensitivity, accuracy, and precision, further supported by cross-validation approaches.

## 7. Label-Free Microfluidic Devices for Exosome Isolation

Even though exosomes (EXOs) stand out as ideal biomarkers for cancer detection and prognosis, their application in clinical routine is not yet a reality. Currently, the purification of EXOs from biological fluids represents a bottleneck for their clinical translation. Among several methods for isolation, differential ultracentrifugation remains the gold standard technology so far. Ultracentrifugation is expensive and time- and labor-consuming, requiring repeated centrifugation steps and large sample volumes. Furthermore, the high centrifugal force may impair EXO morphology and/or promote vesicle aggregation, compromising downstream analyses. Over the past decade, the development of various microfluidics-based devices has paved the way for high yield, easy, and low-cost EXO isolation. Most of these require additional reagents/labels (e.g., immunoaffinity-based isolation), achieving high capture yields but suffering from drawbacks such as low purity and reproducibility. Further information regarding this type of device can be found in some recent excellent reviews [23,87,88]. Conversely, in the present review, we emphasize microfluidics devices that do not require specific particle labelling. These label-free devices are highly attractive; they minimize the cost and complexity of the separation and preserve the EXO structure and composition for downstream analyses. Several strategies have been investigated to achieve label-free particle isolation (Table 6): (i) microfluidic manipulation; (ii) combining microfluidics with electrophoresis (electrofluidics); and (iii) combining microfluidics with acoustic phenomena (acoustofluidics).

### 7.1. Microfluidics-Based Devices

**Figure 7:**
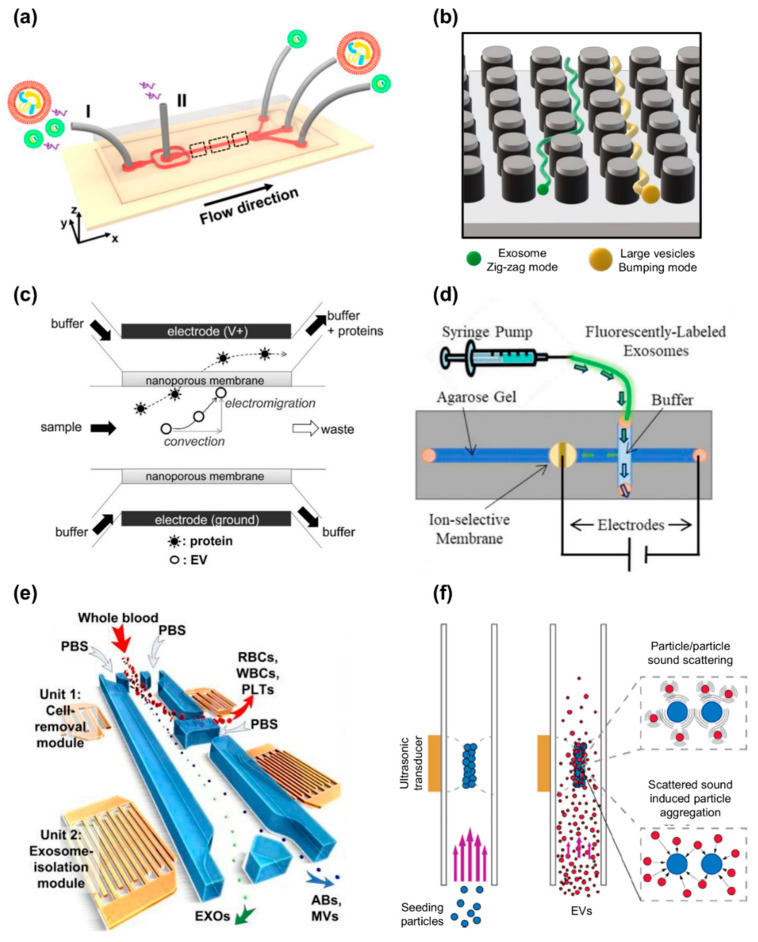
Label-free nanodevices for exosome isolation. (**a**) Schematic illustration of the microfluidic device developed by Liu et al. which exploits lifting forces to separate EVs in a size-dependent manner. Adapted with permission from [199]. Copyright (2017) American Chemical Society. (**b**) Schematic illustration of the microfluidic device realized by Wunsch et al. that uses nanopillar arrays to separate EXOs and other vesicles according to their size [200]. (**c**) Schematic illustration of the microfluidic device presented by Cho et al., which isolates EVs directly from biological fluids by means of electrophoretic migration. Reprinted with permission from [202]. Copyright (2016) Elsevier. (**d**) Schematic illustration of the fluidic device developed by Marczak et al., which uses high transverse electric fields and ion-depleting cation-selective membrane, placed in an agarose gel, to separate EVs of different sizes. Reprinted with permission from [203]. Copyright (2018) John Wiley and Sons. (**e**) Schematic illustration of the acoustofluidic device realized by Wu et al., which exploits SSAW for EXO isolation directly from undiluted blood. Reprinted with permission from [205]. Copyright (2017) National Academy of Sciences. (**f**) Schematic illustration of the acoustic trapping method presented by Ku et al. that exploits ultrasonic transducers to induce reversible clustering between EVs and polystyrene beads which, in turn, facilitates their purification. Adapted with permission from [207]. Copyright (2018) American Chemical Society.

Microfluidics devices are equipped with microchannel and microchambers suited to manipulating fluid flows and affecting the behavior of submicron particles, conferring several advantages such as low sample volume, minimal reagent waste, and short processing time [23,151,208,209,210,211]. For instance, Liu et al. [199] developed a viscoelasticity-based microfluidics device to isolate EXOs from cell culture media and serum (Figure 7a). Viscoelastic manipulation of micrometer particles relies on their migration under the influence of elastic lift forces in a continuous flow. Although this method has previously been applied for the separation of different particles (i.e., blood or tumor cells, bacteria and microspheres), the highest elastic lift forces required for vesicles sorting limit its applicability for EXO isolation. This limitation was overcome by Liu et al. through the addition of poly-(oxyethylene) to the sheath fluid, increasing its viscoelasticity and allowing the generation of sufficient lift forces. The device comprises two inlets, one each for the sample and sheath fluid. After the introduction of the sample at inlet I, the vesicles migrate along the channel sidewalls. Then, the sample fluid intersects the sheath fluid, which is introduced at inlet II, thus altering the alignment of vesicles in a size-dependent manner. Large vesicles are subjected to higher lift forces and therefore are deflected faster to the channel midline and collected at the center outlet. In contrast, smaller vesicles (such as EXOs), which migrate more slowly, remain at the channel sidewalls and are collected at the side outlets. The viscoelasticity-based microfluidics device was able to isolate EXOs from fetal bovine serum with outstanding recovery (93%) and purity (96%). Furthermore, the high processing speed (200 µL h^−1^) guaranteed EXOs integrity after purification. Another microfluidic approach to separate particles by hydrodynamic constraints is nanoscale deterministic lateral displacement (nano-DLD). Nano-DLD is a method that relies on the precise arrangement of an array of nanopillars within a microfluidic channel to manipulate the particle’s trajectory and enable size-dependent separation. The gap length between the nanopillars and the lateral shift of each nanopillars’ row define a critical diameter (D_C_). Particles smaller than D_C_ follow the laminar flow in a “zigzag mode”, whereas particles larger than D_C_ are laterally deflected through the array of nanopillars, following a “bumping mode”. Wunsch et al. [200] manufactured nano-DLD arrays of uniform gap and lengths of 235 nm for the sorting of nanometer particles (Figure 7b). They performed double-stage lithography to define the microfluidic channels and, next, to pattern the nanopillars. The channel at the outlet was designed with a dividing wall that separated the outgoing particles: (i) small particles, which follow a “zig-zag mode” or partially “bumped mode”, are collected at the bottom outlet of the channel; (ii) large particles, which follow a fully “bumping mode”, are deflected and collected to the right outlet; (iii) waste fluid exits via the left-side outlet. With this setup, Wunsch et al. were able to isolate EXOs smaller than 100 nm from human urine, providing an ideal method to separate and analyze EXOs, because it is fast and non-destructive. However, the flow rate with a single nano-DLD array is extremely low (0.1–0.2 nL min^−1^); thus, the device is required to operate constantly for several days. Unlike previous studies, the research group of Demirci [201] realized a microfluidic device designed for the separation of EXOs through a simple filtration approach. The EXO total isolation chip (ExoTIC) relies on a polyethersulfone (PES) filter (pore size of 200 nm) and a low protein-binding filter membrane made from track-etched polycarbonate (30, 50, 80 or 100 nm pore size). The membranes are assembled in a plastic case secured with metal screws and gaskets to prevent fluid leaks. The device can be used standalone or as a modular device, connecting chips with different membrane pore sizes, especially useful for heterogenous particle solutions. The sample is continuously introduced in the ExoTIC chip through a syringe pump at a constant flow rate (5 mL h^−1^); after the filtration, the sample can pass on a chip with smaller pore sizes (as in the case of modular device) or be collected in the same tube. The ExoTIC chip can isolate extracellular vesicles (EVs) from several biological sources (such as plasma, urine and lavage) with higher yield and purity than ultracentrifugation, supporting the device’s wide applicability. This technology meets the ASSURED (affordable, sensitive, specific, user-friendly, rapid and robust, equipment-free, and deliverable to end-users) criteria for disease diagnostics, as stated by the World Health Organization. Specifically, the cost of a single ExoTIC chip is less than USD 1, and could drop even more with large-scale production.

### 7.2. Electrofluidics-Based Devices

Electrofluidics concerns electric fields—matter interactions in microfluidic channels for lab-on-a-chip applications. Cho et al. [202] presented a fluidic chamber for EV isolation directly from biological fluids, which relies on electrophoretic migration (Figure 7c). The device contains three channels (the main one for sample solution and the others for anionic and cationic buffers), two electrodes, and two electrophoretic membranes, with a cut-off size of 30 nm. Two electrodes surround the nanoporous membranes that, in turn, contain the UV-curable epoxy resin main channel (0.9 mm in thickness). A spacer is inserted between each electrode and the adjacent membrane, so that the electrode functions as both a channel wall and an ionic current collector. An electric field transverse to the main channel flow is used to capture EVs and wash nanosized impurities. EVs and most of the plasma proteins have a negative surface charge; therefore, they move toward the anode and encounter the membrane. Only proteins that are smaller than pores (30 nm) pass through the membrane and are flushed out through buffer channels. Conversely, EVs are trapped on the membrane surface, and subsequently can be eluted by pipetting PBS solution. The device is designed to work with a few hundred microliters of plasma, has a capture yield of 60–80%, and operates at a flow rate of 5–40 μL min^−1^. Notably, the system relies solely on physical interactions (electrical migration and size exclusion), thus strongly limiting detrimental effects on sample integrity associated with chemical and biological contaminants. As such, the chip appears to be highly attractive in the sample pre-processing steps. Similarly, Marczak et al. [203] developed a microfluidic electrophoresis device to isolate and concentrate EXOs. The device is composed of a main microfluidic channel and an auxiliary channel transverse to the first. The main channel is filled with agarose gel and an ion-depleting cation-selective membrane is placed at its center. The cation depletion caused by the membrane is exploited to generate a high transverse local electric field. As depicted in Figure 7d, the sample is injected in the auxiliary channel, thus reaching the intersection point. The electric field deflects the particles in the main channel through the agarose gel. The gel pore size (ranging around 200–300 nm) precludes the transit of large particles (such as cells) which flow out of the device, while smaller particles (such as EXOs) migrate towards the membrane. The negative charge of the membrane prevents EXOs (reported zeta potential = −12 ± 3 mV) to cross it; thus, they are trapped within the gel accumulating at the membrane surface. Lastly, the agarose gel is removed and EXOs are recovered. Quantitative fluorescence analysis of isolated EXOs revealed a recovery rate higher than 70% (collection time = 20 min; flow rate = 150 µL h^−1^; field strength = 100 V cm^−1^). The authors confirmed significant EXO isolation and enrichment of the recovery sample through NTA. Pre-concentration of EXOs may be a crucial feature for downstream analysis; however, the presence of an MV population in the recovery sample suggests further optimization of the device. As well as methods that rely on traditional electrophoresis, the research group of Esfandiari [204] realized an insulator-based dielectrophoretic (iDEP) device. The DEP phenomenon is the application of a force that occurs when dielectric particles, under the influence of a non-uniform electric field, are polarized and begin to move. The force intensity depends on several factors (i.e., particle size and shape, particle and medium dielectric properties and frequency field) that can be adjusted to separate different particles. The iDEP device comprises four parallel borosilicate micropipettes enclosed in four pairs of polydimethylsiloxane (PDMS) chambers. The chamber enclosing the tip side of micropipettes is filled with 50 μL undiluted biological fluid, while the chamber enclosing the rear side is filled with 50 μL PBS. The conical geometry of the tip pores (diameter = 1 μm) enables the induction of a strong non-uniform electric field despite a low electric field being applied (∼10 V cm^−1^). The resulting DEP force is balanced by electro-osmosis and electrophoresis forces, thus creating a region near the tip where EXOs are trapped. The iDEP device successfully isolated EXOs from several sources (i.e., plasma, serum, saliva, and cell culture media) from 200 μL samples within 20 min. Moreover, NTA results showed that EXOs isolated through this device are more concentrated than EXOs isolated with ultracentrifugation by two orders of magnitude.

### 7.3. Acoustofluidics-Based Devices

Acoustofluidic devices exploit acoustic radiation forces to separate small particles from large particles. The acoustic force is generated by a standing surface acoustic wave (SSAW) field, often normal to the microfluidic channel. The acoustic force is proportional to the particle volume, while the Stokes drag force, which hinders the particle’s motion, is proportional to the particle radius. Especially for large particle, device operations occur under a regime dominated by acoustic forces. The larger the particle, the greater the difference between acoustic and viscous force. Therefore, large particles (e.g., cells) will move faster toward the acoustic pressure nodes than smaller particles (e.g., EXOs), for which the drag force counters a considerable portion of the acoustic force. This provides the possibility to finely manipulate the cut-off size of particles by setting the suitable acoustic power. For instance, Wu et al. [205] developed an acoustofluidic platform that can isolate EXOs straight from undiluted blood samples (Figure 7e). The device is composed of two tilted-angle SSAW modules arranged in series, each formed by one pair of interdigitated transducer electrodes (IDTs). The first module (cell-removal module) was set at 40 Vpp (peak-to-peak voltage) and a 19.6 MHz driving frequency to remove blood cells (i.e., red and white blood cells and platelets) or, basically, particles larger than 1 µm. The second module (EXO isolation module) was set at 45 Vpp and 39.4 MHz to deflect particles larger than 140 nm to the waste outlet. Overall, the EXO isolation method reported in this work is encouraging, showing purity of 98.4% and a recovery rate of 82.4%. However, as the authors pointed out, a solution of EVs smaller than 140 nm may contain non-exosomal vesicles or protein aggregates, impairing the downstream analyses of EXOs. To tackle this issue, the authors plan to incorporate an additional module into the device to further isolate EXOs by smaller particles and comparable-size vesicles. Lee et al. [206] took advantage of the same principle to realize an acoustic nano-filter microfluidic system for MV and EXO isolation. They designed the device to exert maximal acoustic force on vesicles (>0.1 pN on 1 μm vesicles), enabling size-tunable separation of the latter. The signal driving frequency for SSAW generation was 38.5 MHz. The IDT electrodes were patterned on a PDMS/LiNbO3 piezoelectric substrate and then permanently bonded to the microfluidic structure. The microfluidic device had one center channel for the sample and two sheath channels for the sheath fluid. Under the pressure of the SSAW field, large particles moved faster to the sheath and were collected at the side outlets. Conversely, small particles were collected at the center outlet. The separation was obtained in a continuous and label-free manner. The validation of the device performance was achieved by EXO purification from a complex vesicle solution. EXOs and MVs were isolated with conventional methods (serial filtration and ultracentrifugation), labelled with different fluorophores, and then mixed and processed by the device. Following the separation, the recovery rate was assessed by fluorescence measurements, with reported values of recovery >80% for EXOs and recovery >90% for MVs. Moreover, Western blotting and immunofluorescent microscopy were performed, confirming the exosomal enrichment of the sample eluted in the center outlet. In contrast to the aforementioned studies, Ku et al. [207] presented a non-contact acoustic trapping method based on the application of a secondary acoustic force (Figure 7f). Notably, this method exploits ultrasonic transducers (operating at approximately 4 MHz, 10 V peak-to-peak sinusoidal wave) to apply a primary acoustic force to trap seeding particles (micrometer polystyrene beads) in a borosilicate microfluidic channel. Following this step, the sample containing EVs is added to the channel and a secondary acoustic force enables sound scattering between EVs and the polystyrene beads, inducing particle aggregation. Finally, the resulting clusters are released by turning off the acoustic wave. EV isolation can be achieved from a sample volume of less than 300 μL, with only 30 min of operating time. Acoustic trapping was used to isolate EVs from cell culture media, human urine and plasma. Interestingly, trapped vesicles were much smaller (exosomal size range) than vesicles isolated through ultracentrifugation. Despite the capture efficiency for small beads (100–200 nm) being extremely low (1–5%), Ku et al. demonstrated that it is sufficient for downstream analysis, because EXOs are abundant in biological fluids. Exosomal markers and miRNA expressions of EXO-enriched samples were analyzed. Notably, samples enriched by both acoustic trapping and ultracentrifugation showed similar expressions of exosomal miRNA and tetraspanins such as CD9, CD63 and CD81. Acoustic trapping may pave the way to easy, automated, low-volume compatible and rapid EXO enrichment of biological samples, with no impact on downstream analyses.

## 8. Discussion and Perspectives

In terms of their easy accessibility and capability of representing their parental cells, exosomes (EXOs) and extracellular vesicles (EVs) possess great potential as cancer biomarkers in personalized medicine, playing a key role in emerging liquid biopsy techniques. The clinical potential of these molecules is further confirmed by the impressive growth of publications concerning their diagnostic use. In this context, a wide number of possible EXO biomarkers for different types of cancer have been discovered so far, including prostate adenocarcinoma [212], melanoma [213], pancreatic cancer [214], lung cancer [215], pancreatic ductal adenocarcinoma, and breast cancer [216]. Very interestingly, it was also demonstrated that the EXO-specific molecular cargoes change during cancer evolution [217], enabling discrimination among the various stages of the disease. The latter feature is extremely important, because it extends the spectrum of EV applications in diagnostics, which is not limited to hardly accessible tumors, but includes all types of cancers for diagnosis, staging, and monitoring purposes.

Despite this potential, EV diagnostics are still slowly being accepted by the public, regulatory agencies, and investors and, thus, to be integrated into long-term clinical strategies [218]. Several reviews and position papers in the literature have highlighted the open challenges that need to be faced to stimulate the EXO translational process to the clinic [6,218,219,220,221,222,223]. Based on these papers, we feel that an agreement exists that standardization is the priority line of action. In this regard, the International Society for Extracellular Vesicles has provided guidelines and recommendations to improve result reproducibility, thus boosting the acceptance of the discovered EV biomarkers and improving the communication among scientists with different backgrounds, as well as between academia and industry [218,223,224]. Standardization will deeply impact several aspects of EV science, including sample collection and processing, and EV separation and enrichment. In this framework, a notable challenge lies in the development of sterile, scalable, reproducible, and efficient protocols for clinical-grade EXO production with minimal batch-to-batch variation [225,226]. Albeit particularly relevant for therapeutics, the latter type of development appears to also be extremely useful for diagnostics.

Standardization will not only impact separation, but also EV characterization, and vice versa. EV characterization by multiple, complementary techniques is indeed crucial to assess the results of the diverse separation methods and to establish the likelihood that a biomarker/function is associated with a given EV population. For this purpose, widely used characterization techniques include dynamic light scattering, nanoparticle tracking analyses, tunable resistive pulse sensing, flow cytometry, AFM in the imaging mode, and electron microscopy [219,223]. Concerning EV characterization, the adoption of the label-free techniques discussed here might represent a cornerstone, because these methods contribute to streamlining workflows and reducing/eliminating pre-processing steps which are costly, time-consuming, and can potential alter, degrade or contaminate samples [23].

Here, we have discussed the emerging role of three classes of biophysical techniques, namely, SAS and diffraction, vibrational spectroscopies, and nanoindentation. These techniques are currently widespread among scientists and clinicians working in fields other than EV science. In this regard, we feel that this review fits with one of the key challenges needed to accelerate the maturation of EV studies, which is to facilitate communications among scientists with different backgrounds and specializations [218]. The reviewed literature clearly shows that these techniques are perfectly suited for label-free use, produce quantitative results, and can be successfully combined with nanostructured materials to enhance sensitivity. Most importantly, these techniques provide complementary information compared to conventional characterization methods, such as ELISA, Western Blotting, and omics approaches, thus facilitating the discovery of novel and unexplored cancer biomarkers. Additionally, these techniques could have a pivotal role in the process of standardizing EV samples, because they provide advanced and comprehensive structural, compositional and mechanical information, which is not readily available from most conventional EV characterization techniques.

More specifically, the recent literature shows that SAS and diffraction have the unique capability of providing detailed structural information on nanoscale lipid arrangements in the EV membrane [121,122,123] and its interaction with other nanosized objects [124]. Interestingly, this piece of information reflects the state of the parental cell and its biochemical machinery, being a potential source of clinical valuable information. For instance, this was effectively proven by Accardo et al. [122], who used X-ray lamellar peaks in EXO aggregates to discriminate between cancer and healthy patients, or by Romancino et al. [123] who showed how the inhibition of specific PMTs alters the nanoscale arrangement of EV lipids. Notably, a more in-depth investigation of EV long-term stability has recently been indicated as a key challenge in EXO therapeutics and diagnostics [219,227]. SAXS appears to be exceptionally suitable for investigating this aspect, because it combines large statistical sampling with structural information down to the sub-nanometer level. Due to the latter feature, SAXS will likely outperform electron microscopy and gel electrophoresis for this task, considering that it also does not require sample preparation and that EVs can be measured in a liquid environment [121].

Apart from structural features, a fast and informative label-free biochemical characterization of EVs can be obtained with vibrational spectroscopies, such as Raman and FTIR spectroscopies, which appear to be particularly mature for this purpose, as confirmed by the increasing number of published papers on the subject. Notably, many of these studies discuss preclinical applications in cancer diagnostics, showing the possibility to discriminate EXOs obtained from cancer patients/cells and controls [135,136,139,141,142,146,156,157,160,163]. These methods are particularly intriguing for several reasons, because they are quantitative, easy to use, reproducible, non-destructive, require little or no pre-processing, provide direct access to the specific absorption bands of EV biomolecules, and allow for the development of ultrasensitive applications in combination with nanostructured surfaces or plasmonic nanomaterials [145,146,159,228,229]. An additional characteristic makes Raman and FTIR spectroscopies attractive in EXO diagnostics, and it concerns data analysis. The interpretation of spectral data is often complex, relies mostly on peak assignment, and requires trained personnel capable of removing interferents and recognizing artefacts. To overcome this limitation, spectral data can be analyzed with machine learning techniques that provide physicians with direct diagnostic information through a supervised or unsupervised sample classification. In this context, the reviewed literature shows a frequent application of PCA–LDA or clustering techniques carried out on the acquired spectra or their first derivatives [129,130,135,139,141]. Finally, these spectroscopies allow scientists to access information which is not readily available to other techniques, because they are sensitive to the biomolecule conformations and environments, and can accurately measure the ratio of molecular classes, such as the lipid/protein ratio, which can be lost in omics approaches because of complex sampling techniques. This information will not only stimulate the discovery of disease biomarkers, but also the assessment of long-term EV compositional stability and optimal storage conditions.

Cancer diagnostics has historically been based on the observation of genetic, biochemical, and morphological alterations in cells, the extracellular matrix, tissues, and organs. Aside from these classes of biomarkers, a fourth actor has emerged in the last decade, from the understanding that these alterations are associated with mechanical changes. AFM nanoindentation is one of the most widely used techniques to detect and quantify these mechanical changes at the nanoscale level, with hundreds of applications in diverse fields which range from the analysis of cell biomechanics to the classification of tissue biopsies [170,175,230,231]. Interestingly, mechanical differences in EXOs and MVs reflect changes in cell biomechanics and in cell type, state, treatment, and phenotype. This is not surprising; cell mechanics is mostly governed by the expression and the organization of cytoskeletal proteins that, in turn, play a key role in membrane budding during MV secretion (see Table 1, Section 2). Currently, EV biomechanics is still in its infancy, and most papers on the subject are focused on methodological aspects. This is partly because AFM is an extremely versatile technique, which is a blessing for many research aspects, but it is also a curse when it comes to the standardization and reproducibility of the results. In this context, it is worth recalling the recent paper of Vorselen et al., which detailed an effective protocol for the AFM mechanical characterization of EVs, covering a broad range of aspects, such as EV adhesion to the substrate, data acquisition, quality control, data analysis, and statistical consideration [174]. Interestingly, this protocol allowed the authors to successfully discriminate EVs obtained from patients in different clinical conditions [192]. A second article worth mentioning in this conclusive section was recently published by the group of Valle and co-workers, and aimed to resolve a well-known AFM bottleneck, especially concerning EXOs. AFM indentation is inherently time-consuming and requires specialized personnel with a physical and mathematical background. As such, adapting this technique for high-throughput clinical applications is a challenging task. Starting from the understanding of this issue, the authors developed the first high-throughput method for the characterization of EV mechanics, which is mainly based on an AFM measure of the contact angle and is suited for automated data analysis [193]. To further stimulate the translation process of AFM in diagnostic and liquid biopsy, an additional consideration is needed, regarding the language used to discuss the results. AFM with indention capability is rarely known among clinicians, although it is widely used in base research laboratories devoted to physical, chemical, and material science. Consequently, AFM mechanical biomarkers are poorly accepted and understood by clinicians. One of the reasons for this might be that AFM data analysis is usually not discussed in terms of widely adopted statistical metrics in laboratory medicine, such as ROC curves, sensitivity, specificity accuracy, or, when applicable, survival curves [172,198,232].

A further step needed to stimulate the diffusion of EXO-based liquid biopsies in clinics is the development of advanced purification techniques, capable of replacing conventional extraction methods, such as ultracentrifugation. In this context, label-free microdevices promote a giant leap forward, because they are cheap, easy to use, and suitable for mass production with ultra-large-scale integrated (USLI) technologies. We have highlighted three classes of label-free microfluidic devices, which rely on acoustofluidics, electrofluidics, and size separation with hydrodynamic forces. The reviewed literature provides compelling evidence that these methods not only provide high-purity samples and are capable of working with small amounts of fluid, but are also perfectly suited to work with complex biofluids, such as blood. The above-mentioned characteristics make these microdevices perfectly suited for purifying exosomes for downstream analysis with the label-free characterization techniques discussed above. As can also be inferred from the excellent recent review by Sahoo and collaborators [222], the direct comparison between these devices and conventional isolation methods is not straightforward, because advantages and limitations depend on the specific device working principle. As a general comment, this approach for EXO isolation and enrichment is endowed with exceptional versatility, because custom devices can be designed for a wide range of specific purposes. Moreover, these devices can host sensing elements empowered with nanostructured materials, allowing for simultaneous EXO separation and characterization.

To conclude, we would like to stress that all the methods described in this review can be also effectively combined with labeled techniques in case additional specificity is needed.

## Figures and Tables

**Table 1:** EV classification and biogenic mechanisms.

	Exosomes (EXOs)	Microvesicles (MVs)	Apoptotic Bodies (ABs)
**Diameter (nm)**	30–150	100–1000	100–5000
**Biogenesis**	Budding from endosome lumen (Figure 2B)	Budding from the plasma membrane (Figure 2C)	Released during apoptosis (Figure 2D)
**Biogenesis Steps**	Endocytic materials are internalized and stored in early endosomes; During the maturation of early endosomes in late endosomes, molecules are sorted into intraluminal vesicles (ILVs) with the aid of the ESCRT complexes; Late endosomes can be degraded or fuse with the plasma membrane and release their content, the EXOs, in the extracellular milieu.	The plasma membrane is actively maintained in a state of asymmetry in terms of lipid composition; Calcium-dependent membrane proteins alter the membrane asymmetry leading to the loss of membrane–cytoskeletal anchorage and the formation of regions prone to form blebs; MVs bud from the plasma membrane via ESCRT machinery or through the activation of the ARF6.	Cellular stress, infection, or DNA damage can trigger apoptosis; During apoptosis, a cell undergoes cytoskeleton disruption, nuclear fragmentation, and membrane blebbing releasing ABs; Clearance of apoptotic cells or ABs is operated by professional phagocytes or by neigh-boring cells.

**Table 2:** Summary of selected papers in the literature studying EV structure with SAS and diffraction techniques.

Paper	Aim	Sample/Extraction	Technique (Q-Range nm^−1^)	Model	Main Findings
Varga et al., 2014 [121]	Investigating biophysical properties, i.e., shape and size distribution, of EVs isolated from erythrocytes.	Erythrocyte-derived EVs/RBCs were removed by 2 centrifugation steps at 1550× *g*, t = 20 min and 20 °C. Next, the supernatant was centrifuged (18,890× *g*, 30 min) to concentrate EVs	SAXS (0.015–2.5)	Scattering intensity comprises three contributions: EV scattering modelled with a core–shell form factor weighted with a log-normal distribution; Protein contribution modelled with spherical form factor; Constant background scattering.	Proper modelling of the scattering curve enabled obtaining the size distribution of EVs and discerning EV scattering from contaminants (which can co-precipitate during the purification process).
Romancino et al., 2018 [123]	Exploring the structural arrangement of the lipid bilayer of EV membranes with altered S-palmitoylation state.	EVs secreted by skeletal muscle cells (C2C12 myotubes) at the 3rd day of differentiation (untreated and treated to inhibit S-palmitoylation)/ Ultracentrifugation at 118,000× *g* for 70 min	SAXS (0.03–6.0) SANS (0.05–4.0)	Model-free analysis of SAXS and SANS profiles with neutron contrast variation. Analysis of a hump in the SAXS/SANS scattering profile centered at approximately q = 1.2 nm^−1^, which provide structural information on the bilayer organization (2π/q = 5.2 nm)	SAXS and SANS with neutron contrast variation enables detecting subtle changes in the lipid membrane arrangement in terms of phospholipid head groups and hydrophilic tails associated with the S-palmitoylation state.
Montis et al., 2020 [124]	Studying the interaction between EV-derived supported lipid bilayers (EVSLBs) and gold-coated superparamagnetic iron oxide nanoparticles (SPIONs). Results were compared with artificial SLBs.	EVs secreted by murine prostatic tumor cells (TRAMP-C2 cell line)/ Ultracentrifugation at 100,000× *g* for 240 min	XRR (0.15–0.25) GISAXS (0.15–0.25)	(i.) Model-free analysis of a specific signature of the GISAXS pattern, providing information on the EVSLB/SPION interaction; (ii.) XRR curves were modelled as a multilayer composed of four layers, (i.e., inner polar group, lipid chain, outer polar headgroup, and SPIONs), each characterized by its thickness, scattering length density, and roughness.	As measured with GISAXS, SPIONs are simply absorbed on both SLB surfaces, without membrane/nanoparticle reorganization and thus, without altering membrane biomechanics. A higher absorption is observed on the EVSLBs compared to POCP-SLB, as a consequence of its higher roughness associated with the protein content of exosomes, as measured with XRR.
Accardo et al., 2013 [122]	Classifying exosomes obtained from healthy and cancer cells and concentrated on superhydrophobic patterned surfaces.	Exosomes extracted from two different CCD841-CoN (healthy epithelial colon) cell line and HCT116 (colorectal cancer) cell lines/ ExoQuick Precipitation Solution	WAXS (0.0–3.0) SAXS (0.0–1.8)	Model-free analysis of micro-WAXS/SAXS lamellar peaks in the 3.5 nm^−1^ q range. Micro SAXS patterns measured with benchtop instruments were deionized with a restoration algorithm.	Micro-SAXS/WAXS measurements highlighted differences in the exosome macroaggregates morphology (i.e., number of orders, periodicity, and peak broadening). The authors hypothesized this was due to a more regular organization of exosomes derived from cancer cells than those one extracted from healthy cells, which could be useful to distinguish exosomes with different origins, also for diagnostic purposes.

**Table 3:** Summary of selected papers in the literature studying EV biochemical composition with FTIR spectroscopy.

Paper	Sample/Purification	Methodological Consideration	Main IR Findings	Impact and Application
**Baddela** **2015** [132]	Buffalo’s Milk/ Exoquick	Samples were collected from 3 healthy buffaloes. Band assignment was carried out after averaging 3 spectra.	IR spectra display peculiar absorption bands reflecting exosome composition: (i) 1300–1700 cm^−1^ (amide I–III) and 2700–3500 cm^−1^ (CH stretching) for protein and lipids; and (ii) 900–1200 cm^−1^ for nucleic acids and carbohydrates.	The combined use of IR and miRNA profiles allows for the characterization of bioactive compounds in milk.
**Mihály J.** **2016** [25]	Jurkat T cells/ Centrifugation	Four replicas of the experiment were carried out. The protein–lipid ratio (P/L) was computed as the ratio between the intensity of the amide I–II (1750–1500 cm^−1^) and the CH stretching (3040–2700 cm^−1^). ANOVA was used to compare different EV types	Spectra of EVs and parental cells were compared. ABs’ spectra resemble those of parental cells. The following difference among the diverse EV types were observed in the range 1800–1350 cm^−1^: (i) a shift in the amide I peaks; and (ii) a change in the relative weight of the amide I and II peaks (Figure 5a). The following P/L ratio was measured (Figure 5a): 0.79 ± 0.05 for EXOs; 0.60 ± 0.04 for MVs and 1.20 ± 0.12 for Abs (*P* < 0.0001)	FTIR provides an effective tool for the classification of different EV types. Classification is based on the shape of amide I–II bands and the P/L ratio. These results impact EV sample control, a key issue in exosome science.
**Lee** **2017** [137]	THP-1 cells/ Centrifugation	Three replicates of the experiment were carried out. Comparison among spectra was performed considering the 2nd derivative. PCA loadings were computed to highlight significant spectral changes.	Monocyte activation upon lipopolysaccharide stimulation (LPS) can be inferred from the analysis of released MVs. An increase in the integrated areas of the lipid ester, α-helical protein, and uracil bands upon LPS is observed. Similar spectral changes were detected on monocytes, as confirmed with PCA and PCA loadings.	Spectra of MVs provide biochemical insights into the LPS-induced monocyte model of sepsis. Moreover, IR analysis of MVs is an effective tool to monitor cellular phenotypes.
**Pereira al.** **2018** [138]	CFPAC-1 Cell line and SR4987	Six subjects were recruited for the study, and bone marrow mesenchymal stromal cells were isolated. Spectra were analyzed using the first and second derivatives, and PCA.	The authors studied the influence of culture and time conditioning on exosomes released from human BM-MSCs. Cells were cultured in different media (DMEM and XenoFree). PCA, 1st, and 2nd derivatives showed that IR signatures are more affected by culture conditions than donor or conditioning days.	This paper highlights the role of the different culture conditions in EXO research, showing that great attention has to be paid to this aspect to assure experimental reproducibility.
**Romanò** **2020** [139]	HT29 cells/ Exoquick	Ten replicates of the experiments were carried out. PCA–LDA was used to classify exosomes. PCA loadings were employed to highlight the most relevant spectral changes. Sensitivity, specificity, accuracy, and recall were estimated	The authors studied the biochemical changes in EXOs obtained from HT29 cancer cells under different culture conditions (well-fed and starved cells). Differences in the spectral shape of the amide I–II bands can be used to classify exosomes extracted from the two groups using PCA–LDA. Classification has very high accuracy, precision, and recall, especially in the amide I and II regions.	FTIR combined with PCA–LDA allows for the automated classification of EXOs derived from cells cultured under different conditions. Most importantly, FTIR spectroscopy on exosomes provides information on the cellular state.
**Pascucci** **2014** [134]	CFPAC-1/ Centrifugation	Model-free band assignment (see Figure 5d).	The authors applied FTIR to characterize MVs derived from bone marrow mesenchymal cells. MVs were loaded with PTX, an anticancer molecule. Drug loading induces changes in MV spectra between 3000 and 2800 cm^−1^. These spectral changes show specific features observed in the PTX spectra.	Label-free characterization of EVs with FTIR can provide a quick and effective way of controlling exosome-based nano-cages for drug delivery applications.
**Zlotorogski-Hurvitz** **2019** [136]	Saliva/ Centrifugation	A total of 21 patients diagnosed with oral cancer (OC) and 13 donors (D) were recruited. Machine learning (ML) techniques (PCA–LDA and support vector machine) were used to classify exosomes. Classification performance was evaluated with ROC curves.	The authors highlighted a significant difference in IR spectra between OC and D at 1072 cm^−1^ (nucleic acids), 2924 cm^−1^ and 2854 cm^−1^ (membranous lipids), and 1543 cm^−1^ (transmembrane proteins). The difference was highlighted through relative intensity ratios. An ML-based classification model showed a sensitivity of 100%, specificity of 89%, and accuracy of 95%.	The paper first validates, in a complex clinical setting, a liquid biopsy approach based on the IR characterization of exosomes.
**Martins** **2020** [135]	Serum/ Exoquick	Two cohorts of patients were recruited, with a total of 21 AD patients and 21 controls. The 2nd derivative of FTIR spectra was calculated and a multivariate (PCA, LDA and QDA) and univariate (Mann–Whitney test) analyses were carried out.	EXOs have higher absorbance than serum spectra in the lipid regions (3000–2800 cm^–1^ and 1483–1423 cm^–1^) and the nucleic acids/carbohydrates regions (1200–900 cm^–1^). A multivariate analysis based on 2nd derivative spectra, PCA, LDA, and QDA shows that serum-derived exosomes have better discriminatory properties than serum. A significant difference among the two groups and in both cohorts is measured at 1064 cm^−1^, a peak assigned to ester C–O–C symmetric stretching of phospholipids and/or ribose C–O stretching (nucleic acids).	A key paper providing clinical validation of an exosome-based liquid biopsy approach for AD diagnosis. This study has wide application in diagnostics, because a blood test for AD is still lacking, despite the large research effort in this field.

**Table 4:** Summary of selected papers in the literature studying EV biochemical composition with RAMAN spectroscopy.

Paper	Sample	Methodological Considerations	Outcomes
Tatischeff, (2012) [161]	EVs extracted by UC from D. discoideum cells during growth and starvation and from human urine.	*Technique*: Raman tweezer microspectroscopy *Sample size*: 10 replicates (cell experiment); 4 donors (urine). *Analysis*: Qualitative differences among spectra.	Raman distinguishes EXOs extracted from cells in different conditions (growth and starvation). Raman allows also for the chemical speciation of human EXOs extracted from urine.
Tirinato, (2012) [146]	EXOs extracted by IK from epithelial (CCD841-CoN) and cancer (HCT-116) human cells.	*Technique*: SERS on SHS. *Sample size*: 50 spectra for CCD841-CoN and HCT-116 cells. *Analysis*: Qualitative differences among spectra.	Raman signal is improved by combining SERS, that enhances the electromagnetic field, and SHSs, that increase EXO concentrations. The method allowed the authors to distinguish EXOs from epithelial and cancer cells.
Kerr, (2014) [156]	EXOs extracted by UC from ovarian carcinoma cells (A2780) in normoxia and hypoxia conditions.	*Technique*: SERS with AuNPs and Raman microspectroscopy. *Sample size*: 10 spectra for each condition. *Analysis*: multivariate (PCA and DFA).	The use of SERS and Raman microspectroscopy (RM) in the diagnostic field was explored. RM outperforms SERS in distinguishing EXOs extracted from the two conditions (normoxia and hypoxia).
Smith, (2015) [162]	EXOs extracted by UC from different cell lines: A549, Huh-7, SKOV3, IMR90, Jurkat, Kasumi-1, and 3T3.	*Technique*: Laser Tweezers Raman Spectroscopy (LTRS); *Sample size*: From 10 to 20 single EXOs measured for each cell line; *Analysis*: multivariate (PCA).	LTRS allowed authors to distinguish EXOs derived from several cell lines and different EXOs subpopulations in the same sample, thanks to the single EXO analysis.
Lee, (2015) [161]	EXOs extracted by total exosome isolation reagent (TEIR) and UC from ovarian cancer cell line (SKOV-3).	*Technique*: SERS with silver-coated nanobowl substrates. *Sample size*: 10 spectra for each time point. *Analysis*: PCA of the Raman spectra.	Lee et al. developed nanobowl SERS substrates that can capture and allow molecular-level EXO characterization. At the start of analysis, SERS spectra exhibited typical lipids and protein peaks. Later, new peaks developed, suggesting ruptures of EXOs over time, enabling the analysis of EXO content.
Stremers(2016) [153]	ELVs extracted by UC from melanoma cancer cells (B16F10) and human RBCs.	*Technique*: SERS with AuNPs. *Analysis*: PLS-DA and MCR-ALS. *Sample size*: 25 (B16F10), 41 (RBCs) and 60–80 spectra for the mixtures.	SERS, in combination with Au nanoparticles, allowed the use of PLS-DA analysis to discriminate spectra between RBC-derived and cancer-derived EXOs.
Gualerzi (2017) [163]	EVs extracted by DC from human bone marrow and adipose tissue mesenchymal stromal cells (MSCs), and dermal fibroblasts.	*Technique*: Micro-Raman. *Sample size*: 10 independent replicates for each cell type. *Analysis*: multivariate (PCA of normalized spectra, LDA classification using the first 25 PCs); univariate (ANOVA on PC scores).	The main outcome of this work is the presented Raman analysis can distinctly discern not only vesicles from MSCs and terminally differentiated fibroblasts, but also vesicles of MSCs from bone marrow and adipose tissue.
Park, J. (2017) [142]	EXOs were extracted by DC and chromatography from human lung carcinoma (H1299, H522) and PAEpiC cell lines.	*Technique*: SERS with AuNPs. *Sample size*: 37 samples of H1299, 34 of H522, and 23 of alveolar cell-derived EXOs. *Analysis*: PCA of the Raman spectra.	SERS measurements and statistical PCA analysis were used to develop a method for the detection of EXOs derived from cancer cells.
Sivashanmugan, (2017) [158]	EXOs extracted by UF from: epithelial (NL-20, Beas-2b), adenocarcinoma (PC9, HCC827 and H197) human cell lines and L929 murine cell line.	*Technique*: SERS on a substrate of Ag nanocubes (NCs) and Au nanorod (NR) array. *Analysis*: Qualitative study of the differences between spectra.	Sivashanmugan et al. developed different substrates for SERS analysis of EXOs. EXOs derived from lung adenocarcinoma cells exhibited a stronger and more heterogeneous signal in the protein band than EXOs derived from normal cells.
Avella-Oliver, (2017) [160]	EXOs extracted by UC from lung cancer cell line (A549 UC).	*Technique*: SERS on a silver cover substrate from a compact disk. *Analysis*: Qualitative observation of the Raman spectra.	Avella-Oliver et al. realized a novel cost-effective substrate for SERS analysis of EXOs based on regular recordable optical disk structures covered with silver.
Shin, (2018) [155]	EXOs extracted by chromatography from NSC lung cancer (PC9 and H1299) and pulmonary alveolar epithelial (HPAEC) human cell lines.	*Technique*: SERS on a substrate of Au nanoparticles coated with cysteamine for in liquid measurements. *Sample size*: 25 spectra for each sample. *Analysis*: PCA and ratiometric analysis.	In this work, Shin et al. showed differences in spectra obtained from NSCLC- device EXOs and HPAEC-derived EXOs. These was compared with the spectra of some protein markers to better understand the changes in cancer derived EXO composition, using both PCA and ratiometric approach.
Yan, (2019) [141]	EXOs extracted by UC and ExoQuick from HCC827 and H1975 lung adenocarcinoma cell lines, FBS and human serum.	*Technique*: SERS on hybrid substrate made of a graphene-covered Au surface containing a quasi-periodic array of pyramid. *Sample size*: 100 spectra for each sample. *Analysis*: PCA.	The method developed by Yan et al. enabled single EXO measurements. Efficient discrimination between EXOs derived from different biological sources was achieved by unbiased PCA.
Kruglik, (2019) [164]	EVs extracted by UC from human urine after an 8 h fasting period and from primary rat hepatocytes with and without Acetaminophen treatment.	*Technique*: Raman tweezer microspectroscopy (RTM) in the near-infrared region. *Analysis*: Biomolecular component analysis based on Raman markers. *Sample size*: 2 donors (urine); 5 and 7 EXO sets, collected from treated and untreated rat hepatocytes, respectively.	Kruglik et al. presented a comprehensive picture of the RTM potentialities and limitations for EXO characterization. The method demonstrates its capacity to unravel the different molecular contribution to EVs (proteins, lipids, nucleic acids, carotenoids, etc.).
Zhang, (2019) [162]	EXOs extracted by UC from esophageal (EC109, EC9706 and Kyse150), breast epithelial (M231 and MCF7), and hepatoma (HepG2) human cell lines.	*Technique*: SERS with AuNPs. *Sample size*: 35 spectra acquired for each cell line *Analysis*: PCA/LDA of the Raman spectra and ratiometric approach.	The application of PCA/LDA algorithm to SERS data allowed the classification of EXOs derived from 8 different sources. The authors also found that the 600–760 cm^−1^ region is associated with great differences in esophageal cells, whereas the 940–1100 cm^−1^ region is associated with breast cells.

**Table 5:** Summary of methodological AFM papers investigating EV biomechanics.

Paper	Sample/Purification	AFM Methods	Mechanical Findings	Impact and Application
**Sharma 2010** [190]	EXOs extracted from Saliva by Ultracentrifugation (UC).	EXOs were absorbed overnight on mica. Measurements were performed in PBS using a soft (k = 0.02 N/m) MSCT cantilever (Veeco) at 0.25 Hz. AFM was used in the amplitude and phase modulation mode. Applied forces ranged from 1.4 nN to 2.4 nN.	EXOs deform under applied forces. Deformation is accompanied by a tri-lobed-shaped depression region in the particle center. The larger the applied force in the 1.4–2.4 nN, the deeper the depression (Figure 6b). Strong specific adhesions forces were measured using CD63-conjugated tips.	One of the first papers focused on EXO biomechanics highlighting their deformation under applied force and pointing out the need to use low forces for imaging purposes. The paper also shows that AFM can be used for specific exosome detection, through tip conjugation, thus being potentially useful in diagnostics.
**Li 2021** [194]	EXOs extracted by UC from the bone marrow of lymphoma patients.	EXOs were absorbed on Poly-L-Lysine-coated slides and characterized with peak force tapping (PFT) AFM in PBS with a silicon nitride tip (k = 0.7 N/m, f = 150 kHz, and tip radius 20 nm).	Poly-L-Lysine facilitates AFM measurements of EXOs improving the height/diameter ratio (~0.3) compared to air-drying. PFT is suitable for the quantitative imaging of EXO topography, stiffness, adhesion and dissipative properties, also highlighting the contrast with the substrate. Dissipation is not homogeneous and symmetric on EXO surfaces.	These results demonstrate that AFM in the PFT mode is suitable for a quick and effective quantitative analysis of EXOs, and thus is of potential use for the search of EXO-based biomarkers.
**Parisse 2013** [195]	SKBR3-derived EXOs.	The IT-AFM experiment was performed in liquid using a cantilever with k = 0.1 N/m and f = 32 kHz.	The typical shape of EXO FD curves is reviewed, highlighting their resemblance with curves acquired on artificial lipid vesicles.	In-depth theoretical models developed for artificial vesicles can be used to analyze extracellular vesicles.
**Vorselen 2020** [174]	EXOs extracted from serum of healthy donors and patients diagnosed with hereditary spherocytosis.	EVs were absorbed on a poly-L-lysine coverslip. Imaging was performed using FD-based AFM with forces < 0.1 nN, reducing EV compression. Mechanics were measured through FD curves at 0.5 nN at a slow speed of 0.2–1 Hz (elastic response). Higher forces were used to penetrate the lipid bilayer and record membrane tethering.	The paper validates a protocol based on the Canham–Helfrich model to decouple EV bending modulus and La Place pressure. The method uses information from indentation and membrane tethering, which provides surface tension, σ. Steps are detailed for measuring σ, a non-trivial task because of tip contamination. The correct FD curve hallmarks are discussed: (i) a smooth indentation between the contact point and the EV height; (ii) an abrupt increase in reaction force when the two bilayers are pressed together; (iii) two discontinuities indicating 1st and 2nd bilayer rupture; and (iv) the interaction with the substrate.	A key paper in EXO biomechanics, as it provides a unified protocol for performing nanoindentation on vesicles and the subsequent data analysis. The paper guides scientists step by step through sample preparation, FD curve acquisition, model choice and application and statistical analysis of the data.
**Ridolfi 2019** [193]	EXOs extracted by UC-SEC from milk and nematodes	EXOs were absorbed on Poly-L-Lysine. Measurements were performed in peak force mode in DI water using Bruker SNL-A probes (tip radius 2–12 nm, k = 0.35 N/m). Applied force was 0.15–0.25 nN, cantilever speed < 5 µm/s	The authors developed an AFM protocol to characterize artificial and natural vesicles mechanics in liquid by AFM imaging: (i) the method allowed them to retrieve EV unperturbed geometry and contact angle α in terms of particle height and surface projected radius; (ii) α depends on stiffness and it is roughly independent of EV size; (iii) a deviation from point ii indicates the presence of contaminants; (iv) a calibration curve is provided that allows calculating stiffness (a mechanical parameter) from contact angle (a morphological parameter).	A potential game-changer in the field, this paper presents the first high-throughput method for EXO mechanics based on imaging. The study derives stiffness from contact angles, and it is based on the understanding that EVs are deformed by adhesion forces into an equilibrium shape that is a direct consequence of EV stiffness. The method also allows the removal of contaminants, a key issue in EXO research.

**Table 6:** Summary of selected label-free devices for exosome purification from culture media and body fluids.

Paper	Sample	Purification Strategy	Fabrication and Materials	Flow Conditions and Yield	Main Findings
C. Liu et al., 2017 [199]	Fetal bovine serum	Microfluidics - Elastic lift forces	Standard soft lithography techniques PDMS	- Flow rate = 200 µL h^−1^ - EXO recovery = 93% - EXO purity = 96%	The microfluidic device can separate EVs in a size-dependent manner. The separation is based on the generation of high viscoelastic lift forces, exerted on vesicles, due to the addition of a polymer (PEO) in the media.
B. Wunsch et al., 2016 [200]	Human urine	Microfluidics- Deterministic lateral displacement	Double-stage lithography (microfluidic channels nanopillars) SiO_2_ hardmask	- Flow rate = 0.1–0.2 nL min^−1^	Wunsch et al. realized DLD arrays of nanopillars (pillar gap sizes ranging from 25 to 235 nm) to separate EXOs smaller than 100 nm from heterogeneous vesicles samples with sharp resolution.
F. Liu et al., 2017 [201]	Plasma, urine and lavage fluid	Microfluidics - Filtration chip	PES filters (200 nm pore size) and low protein binding membranes (track-etched polycarbonate, 30–100 nm pore size), assembled in a plastic case.	- Flow rate = 5 mL h-1 - Least volume = 10−100 μL - Yields 4-to1000-fold higher than ultracentrifugation	The ExoTIC chip can isolate EVs from several biological sources due to a series of filtration membranes with higher yield and purity of standard methods. This technology meets the ASSURED criteria stated by the WHO; especially, the cost of a single ExoTIC chip is less than USD 1.
S. Cho et al., 2016 [202]	Mouse plasma	Electrofluidics - Electrical migration and size exclusion	UV-curable epoxy resin channels, PCTE membranes (30 nm pore size) and disk electrodes.	- Flow rate = 5–40 μL min^−1^ - Capture yield = 60–80% - Least volume = ~500 μL	Cho et al. presented a fluidic chamber for EV isolation directly from biological fluids that rely solely on physical interactions, limiting detrimental effects on sample integrity. Notably, an electric field applied across a dialysis membrane aids protein migration but captures EVs on the membrane surface.
S. Marczak et al., 2018 [203]	Human serum	Electrofluidics - Electrical migration and size exclusion	Microfluidic chip was made from 300 µm polycarbonate sheets. Cation-exchange membrane has sulfonic or carboxylic acid groups that attract cations.	- Flow rate = 150 µL h^−1^ - EXO recovery = 70%	Marczak et al. presented a simple microfluidic device to simultaneously isolate and preconcentrate EXOs by trapping them in agarose gel using an ion-depleting cation-selective membrane. The cation depletion caused by the membrane is exploited to generate a high transverse local electric field.
L. Shi et al., 2019 [204]	Cell culture media, serum, plasma and saliva.	Electrofluidics - Dielectrophoresis separation	Micropipettes were fabricated using the laser-assisted puller. PDMS chambers were fabricated and bonded with a glass slide via oxygen plasma cleaner.	- Least volume = 200 μL - Yield = two orders of magnitude higher than ultracentrifugation	Shi et al. realized an insulator-based dielectrophoretic device based on micropipettes with conical tip pores, thus enabling the induction of a strong non-uniform electric field. The resulting DEP force—balanced by electroosmosis and electrophoresis forces—creates a region where EXOs are trapped.
M. Wu et al., 2017 [205]	Undiluted blood	Acoustofluidics - SSAW	Two SSAW modules arranged in series. The cell-removal module was set at 40 Vpp and 19.6 MHz. The exosome isolation module was set at 45 Vpp and 39.4 MHz.	- Flow rate = 4 μL min^−1^ - EXO recovery = 82% - EXO purity = 98%	Wu et al. developed an acoustofluidic platform for EXO isolation straight from undiluted blood. The device is composed of two tilted-angle SSAW modules arranged in series. The first module removes particles larger than 1 µm (e.g., blood cells), while the second module particles larger than 140 nm.
K. Lee et al., 2015 [206]	Polystyrene beads, cell culture media and RBCs	Acoustofluidics - SSAW	The IDT electrodes for SSAW generation (38.5 MHz) were patterned on a PDMS/LiNbO_3_ piezoelectric substrate and bonded to the microfluidics.	- EXO recovery > 80% - MV recovery > 90%	Lee et al. realized an acoustic nano-filter microfluidic system for MVs and EXOs isolation. They designed the device to exert maximal acoustic force on vesicles (>0.1 pN on 1-μm vesicles), enabling size-tunable separation of the latter in a continuous and label-free manner.
A. Ku et al., 2018 [207]	Cell culture media, human urine and plasma	Acoustofluidics - Acoustic trapping	Automated trapping device, AcouTrap (AcouSort^®^).	- Least volume = 300 μL - Trapping efficiency = 1–5%	Using acoustic trapping, Ku et al. enriched EVs from several biological sources. This method exploits ultrasonic transducers to apply a primary and secondary acoustic force, to first trap seeding particles, and then to induce particle aggregation between EVs and seeding particles, allowing vesicle isolation.

## Data Availability

Not applicable.
